# Treatment-related adverse events of immune checkpoint inhibitors in clinical trials: a systematic review and meta-analysis

**DOI:** 10.3389/fonc.2024.1391724

**Published:** 2024-05-17

**Authors:** Xin Shen, Jun Yang, Geng Qian, Mingyu Sheng, Yu Wang, Guohui Li, Jiaqing Yan

**Affiliations:** Department of Pharmacy, National Cancer Center/National Clinical Research Center for Cancer/Cancer Hospital, Chinese Academy of Medical Sciences and Peking Union Medical College, Beijing, China

**Keywords:** immune checkpoint inhibitor, immune-related adverse events, treatment-related adverse events, cancer, systematic review, meta-analysis

## Abstract

**Aim:**

This study comprehensively assesses the incidence and profiles of treatment-related adverse events (trAEs) of immune checkpoint inhibitor (ICI)-based therapies across cancer at various sites.

**Methods:**

We systematically searched the PubMed, Embase, and Cochrane databases for trials investigating ICI-based therapies published between their inception and August 2023.

**Results:**

In total, 147 studies involving 45,855 patients met the inclusion criteria. Among them, patients treated with ICIs reported 39.8% and 14.9% of all-grade and grade ≥3 immune-related adverse events (irAEs), respectively. The most common all-grade irAEs were dermatological and gastrointestinal issues, diarrhea, and pruritus, whereas patients who received ICIs showed most common grade ≥3 irAEs, including gastrointestinal events, diarrhea, increased aspartate aminotransferase and alanine transaminase levels, and hepatic and dermatological events. The overall trAE incidence in patients treated with ICIs was 83.2% for all-grade trAEs and 38.2% for grade ≥3 trAEs. TrAE incidence was highest for patients treated with cytotoxic T lymphocyte antigen-4 inhibitors for all-grade and grade ≥3 trAEs, with incidences of 86.4% and 39.2%, respectively. ICIs combined with targeted therapy showed the highest all-grade and grade ≥3 trAEs, with incidences of 96.3% and 59.4%, respectively. The most common all-grade trAEs were anemia, decrease in white blood cell count, decrease in neutrophil count, nausea, fatigue, diarrhea, and alopecia; patients who received ICIs presented relatively high incidences of grade ≥3 trAEs.

**Conclusion:**

This study provided comprehensive data regarding irAEs and trAEs in patients receiving ICIs. These results should be applied in clinical practice to provide an essential reference for safety profiles of ICIs.

**Systematic review registration:**

INPLASY platform, identifier INPLASY202380119.

## 1 Introduction

Immune checkpoint molecules play a crucial role in the immune regulation of malignant tumors, and their biological significance is essential for the diagnosis, prognosis, and treatment of tumors (1). Checkpoints are located on various immune cells, including T lymphocytes, or on tumor cells, and they function like switch proteins by inducing various signals to control the excessive activation of T cells. T cell dysfunction may be attributed to continuous antigen exposure and the overexpression of multiple inhibitory receptors, ultimately leading to a decrease in the proliferation or function of T cells in cancer. Immune checkpoint blockade by immune checkpoint inhibitors (ICIs) primarily targets immune checkpoints expressed on the surface of immune cells, and it is a therapeutic approach that enhances the recognition and elimination of tumor cells by the immune system (2). Thus, use of ICIs is considered as a novel treatment strategy for cancer, which can inhibit tumor evasion and enhance the immune response via targeted silencing of cytotoxic T lymphocyte antigen-4 (CTLA-4) and programmed death-1/ligand-1 (PD-1/PD-L1) (3). Studies have demonstrated that targeted immune checkpoints have shown impressive antitumor activity across various types of cancer (4, 5). However, a certain proportion of patients do not respond to ICIs and show immune-related adverse events (irAEs); it is important to address irAEs in clinical practice (6, 7).

Although ICIs have significant benefits in cancer treatment, they can also cause various side effects because of checkpoints are heavily expressed in various organs other than the cancer (8–10). Although the prevalence of most serious adverse events (AEs) is low, they can still be fatal (11, 12). Moreover, considering the response rate to ICIs is important in clinical practice and ICIs combined with targeted therapies or chemotherapy are being widely used. However, there have been increasing concerns regarding the safety of ICI treatment. Furthermore, many patients do not benefit from therapy or even experience multiple irAEs; the side effects of ICIs can be devastating for the immune system and may accelerate disease progression. Thus, ICI safety profiles should be fully elucidated to achieve greater efficacy and minimize AEs.

Several systematic reviews and meta-analyses have investigated ICI adverse effects on cancer at specific sites and found that the use of ICIs could increase the risk of toxicity and treatment discontinuation (13–17). The increased risk of AEs is a challenge in the development of novel ICIs, especially for combined treatments in clinical practice (18). The safety profiles of ICI treatments should be summarized to guide clinicians in balancing the benefits and risks of therapy. Therefore, we performed this study to provide detailed toxicity profiles for ICIs and compare the incidence of AEs according to the types of cancer and ICI.

## 2 Materials and methods

### 2.1 Search strategy and selection criteria

This study was conducted in accordance with the Preferred Reporting Items for Systematic Reviews and Meta-Analyses (PRISMA) guidelines (19). Our study was registered in INPLASY platform (number: INPLASY202380119). Randomized controlled trials (RCTs) applying ICIs to cancer at various sites and reporting treatment-related adverse events (trAEs) were eligible for inclusion. PubMed, Embase, and the Cochrane library were systematically searched for eligible trials throughout August 2023, and the search terms included “immune checkpoint inhibitors” and “randomized controlled trial” (**Supplementary 1**). Trials that had already been completed but not yet published were searched on the https://clinicaltrials.gov website (US NIH). We manually searched the reference lists of relevant reviews and articles to avoid omitting eligible articles.

Two reviewers performed the literature search and selected the studies using a standardized approach, which refers to two authors independently conducting literature screening, followed by cross-checking the screening results. Disagreements were resolved by a third reviewer until a consensus was reached among all three reviewers. The following selection criteria were used: (1) studies designed as RCTs and published in English; (2) trials including patients who concurrently received two categories of treatments, at least one of which was an ICI (ipilimumab, pembrolizumab, nivolumab, tremelimumab, atezolizumab, durvalumab, avelumab, camrelizumab, cemiplimab, tislelizumab, toripalimab, sintilimab, adebrelimab, and sugemalimab); (3) trials reporting tabulated data of irAEs, trAEs, or specific AEs based on Medical Dictionary tor Regulatory Activities; and (4) sample size > 10. Trials that included patients treated with a combination of two classes of ICIs or patients who received sequential combination therapies were excluded. We selected the most recent trials or trials reporting a comprehensive AEs profile if the same population was published more than once.

### 2.2 Data collection and risk-of-bias assessment

A standardized flowchart was applied by two reviewers to extract all relevant information from the included studies, and any inconsistencies between the reviewers were resolved via discussion until a consensus was reached. The following data were collected: first author name, publication year, registered number, country, sample size, mean age, male proportion, cancer type, intervention, combined treatments, and outcomes. The primary endpoints of this meta-analysis were all-grade and grade ≥ 3 irAEs, whereas the secondary endpoints included all-grade and grade ≥ 3 trAEs and the profiles of all-grade and grade ≥ 3 specific AEs. The Cochrane risk-of-bias tool was used to assess methodological quality according to random sequence generation, allocation concealment, blinding of participants and personnel, blinding of outcome assessment, incomplete outcome data, selective reporting, and other biases (biases associated with the research design used, premature termination of the study, significant baseline feature imbalance, presence of deceptive behavior, and other factors) (20). Two reviewers independently assessed the quality of individual trials, and conflicts between the reviewers were resolved by an additional reviewer.

### 2.3 Statistical analysis

Random-effect models with a logit transformation were applied to pool the overall AE incidences and profiles, and restricted maximum likelihood estimation was used to fit all models via a classic continuity correction for zero cells and sample sizes (21). Effect estimates were calculated using incidence with a 95% confidence interval (CI), and a division method was used to calculate the incidence (22). *I^2^* and Q statistics were used to assess heterogeneity, and significant heterogeneity was defined as *I^2^* > 50·0% or *P* < 0·10 (23). Further exploratory analyses were performed to identify whether the incidence of all-grade and grade ≥ 3 irAEs and trAEs differed based on the type of ICI and combination therapy, and the differences between subgroups were compared using the interaction *t*-test (24). Publication bias was assessed using funnel plots and quantified using the Egger and Begg tests (25, 26). The *P* value for the pooled estimates was two-sided, and the inspection level was 0.05. All analyses were performed using the STATA software (version 12.0; Stata Corporation, College Station, TX, USA).

## 3 Results

### 3.1 Literature search and study selection

**Figure 1:**
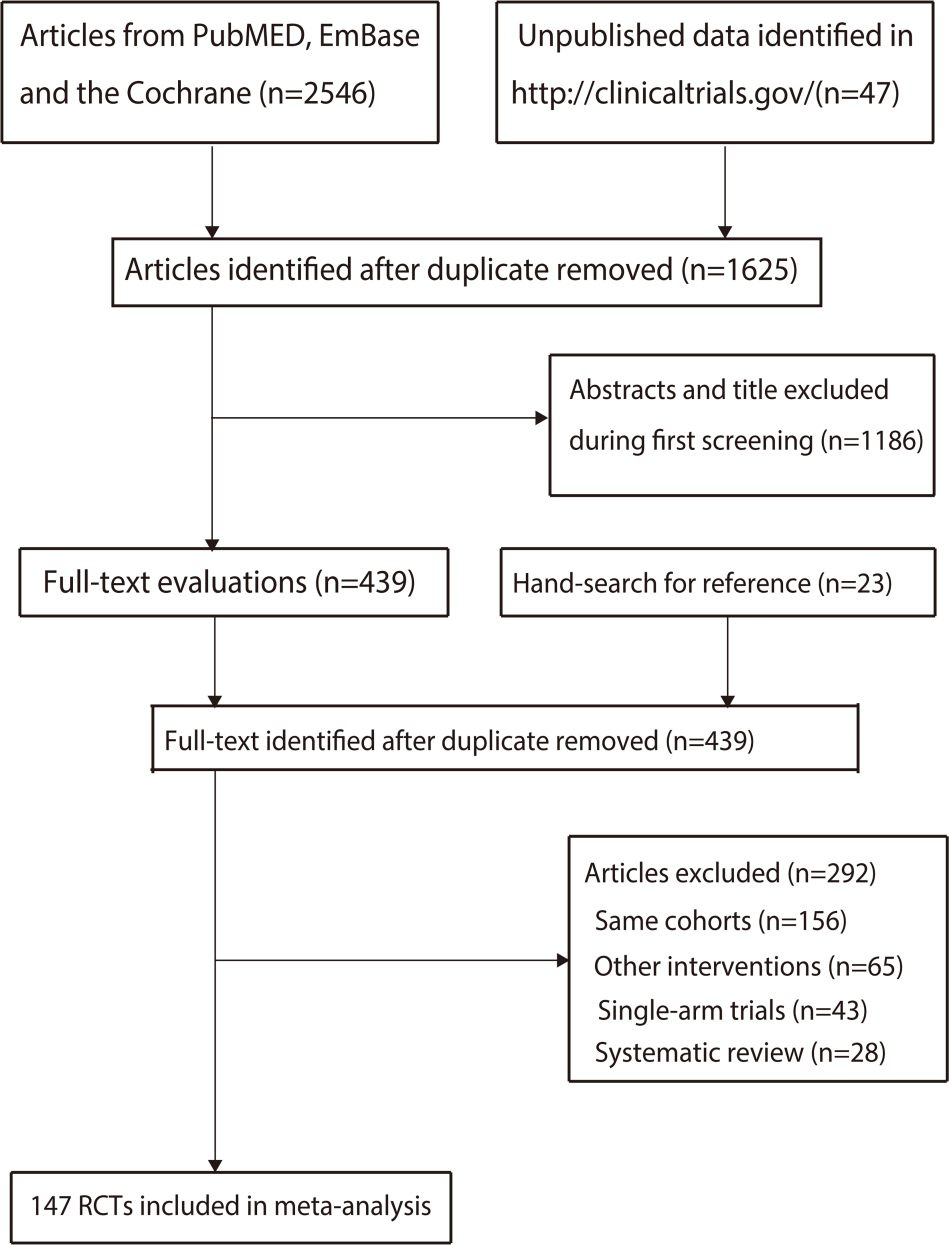
The PRISMA flowchart for trials selection process.

A total of 2,546 publications were identified from the literature searches, and 921 were excluded because of duplication. A further 1,186 articles were excluded because of irrelevant titles or abstracts. The remaining 439 studies were retrieved for full-text evaluation, and 292 were excluded for the following reasons: studies reporting the same populations (n = 156), combining two classes of ICIs (n = 65), single-arm trials (n = 43), and systematic reviews (n = 28). Manual reviews of the reference lists identified 23 articles, all of which were excluded because of duplication. Overall, 147 RCTs involving 45,855 patients were identified between 2010 and 2023, and 14 ICI types were compared in the final systematic review and meta-analysis (**Figure 1**).

### 3.2 Trial characteristics

The characteristics of the identified studies and their patients are summarized in **Supplementary 2**. The sample sizes of the included trials ranged from 13 to 906 participants, and the mean age ranged from 36.0 to 75.5 years. In total, 124 trials were multinational, whereas the remaining 23 were conducted in a single country. The safety profiles of ipilimumab, pembrolizumab, and nivolumab were investigated in 14, 39, and 37 trials, respectively, whereas four, 23, and 10 trials assessed the safety profiles of tremelimumab, atezolizumab, and durvalumab, respectively. Moreover, the safety profiles of avelumab, camrelizumab, cemiplimab, and tislelizumab were assessed in five, five, three, and four trials, respectively, whereas three, five, one, and one trials reported the safety profiles of toripalimab, sintilimab, adebrelimab, and sugemalimab, respectively. **Supplementary 3** presents the quality of the included studies. Although 70 studies reported unclear risk of bias for allocation concealment, and 32 trials reported unclear other biases, the summary risk of bias in all trials were low.

### 3.3 IrAEs

**Figure 2:**
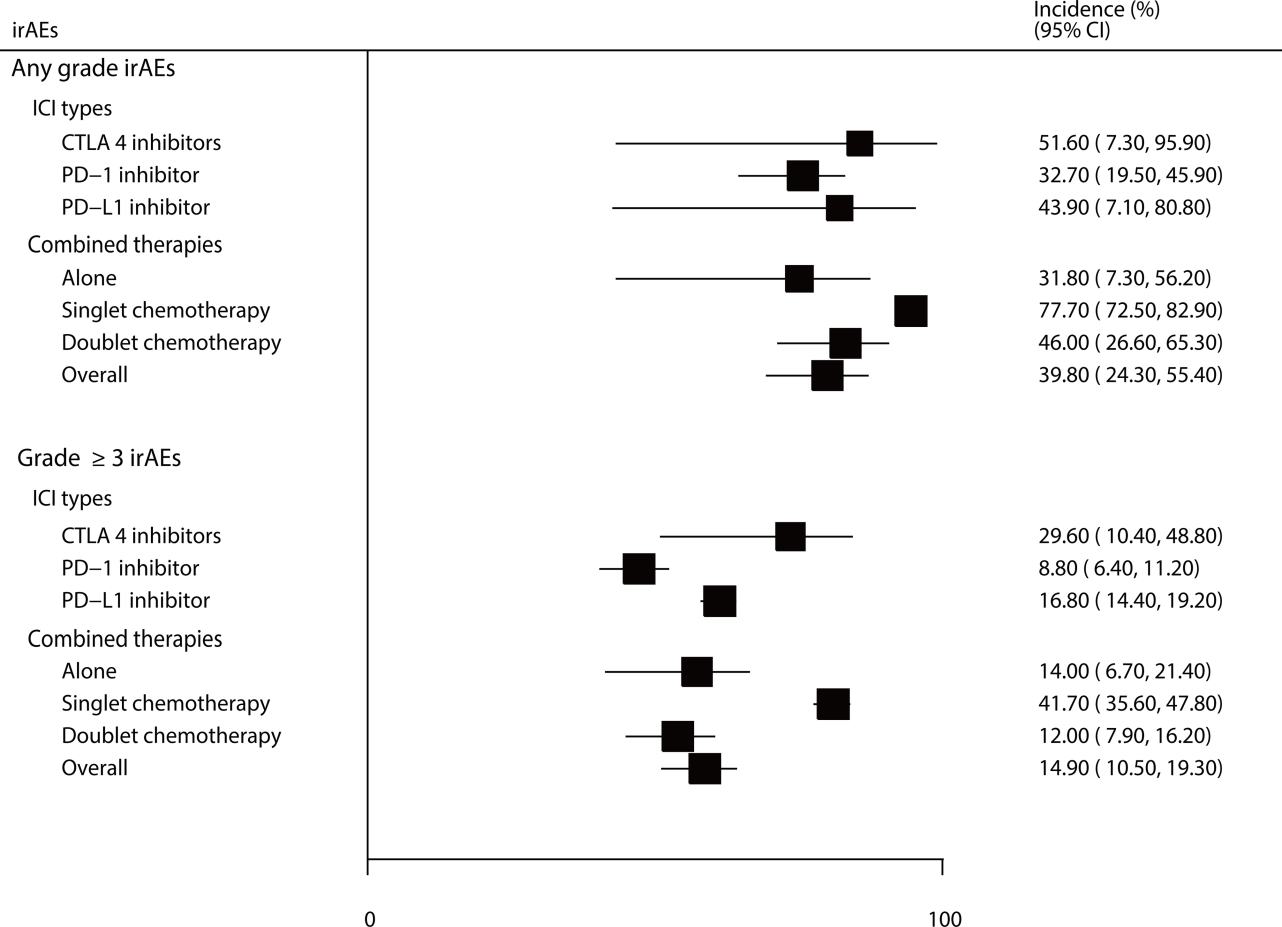
The summary incidences for all-grade and grade ≥ 3 irAEs.

The incidences of all-grade and grade ≥ 3 irAEs were 39.8% (95% CI: 24.3–55.4%) and 14.9% (95% CI: 10.5–19.3%), respectively. Moreover, we noted significant heterogeneity for all-grade (*I^2^ = *99.6%; *P* < 0.001) and grade ≥ 3 irAEs (*I^2 = ^* 96.3%; *P* < 0.001) in patients treated with ICIs. Exploratory analyses were performed to identify potential sources of heterogeneity, and we noted that the incidences of all-grade irAEs for patients treated with CTLA-4, PD-1, and PD-L1 inhibitors were 51.6% (95% CI: 7.3–95.9%), 32.7% (95% CI: 19.5–45.9%), and 43.9% (95% CI: 7.1–80.8%), respectively. For grade ≥ 3 irAEs, these respective percentages were 29.6% (95% CI: 10.4–48.8%), 8.8% (95% CI: 6.4–11.2%), and 16.8% (95% CI: 14.4–19.2%). When stratified by combined therapies, the incidences of all-grade irAEs for patients treated with ICIs alone, combined with singlet chemotherapy, and combined with doublet chemotherapy were 31.8% (95% CI: 7.3–56.2%), 77.7% (95% CI: 72.5–82.9%), and 46.0% (95% CI: 26.6–65.3%), respectively. For grade ≥ 3 irAEs, these incidences were 14.0% (95% CI: 6.7–21.4%), 41.7% (95% CI: 35.6–47.8%), and 12.0% (95% CI: 7.9–16.2%), respectively (**Figure 2**, **Supplementary 4**).

**Figure 3:**
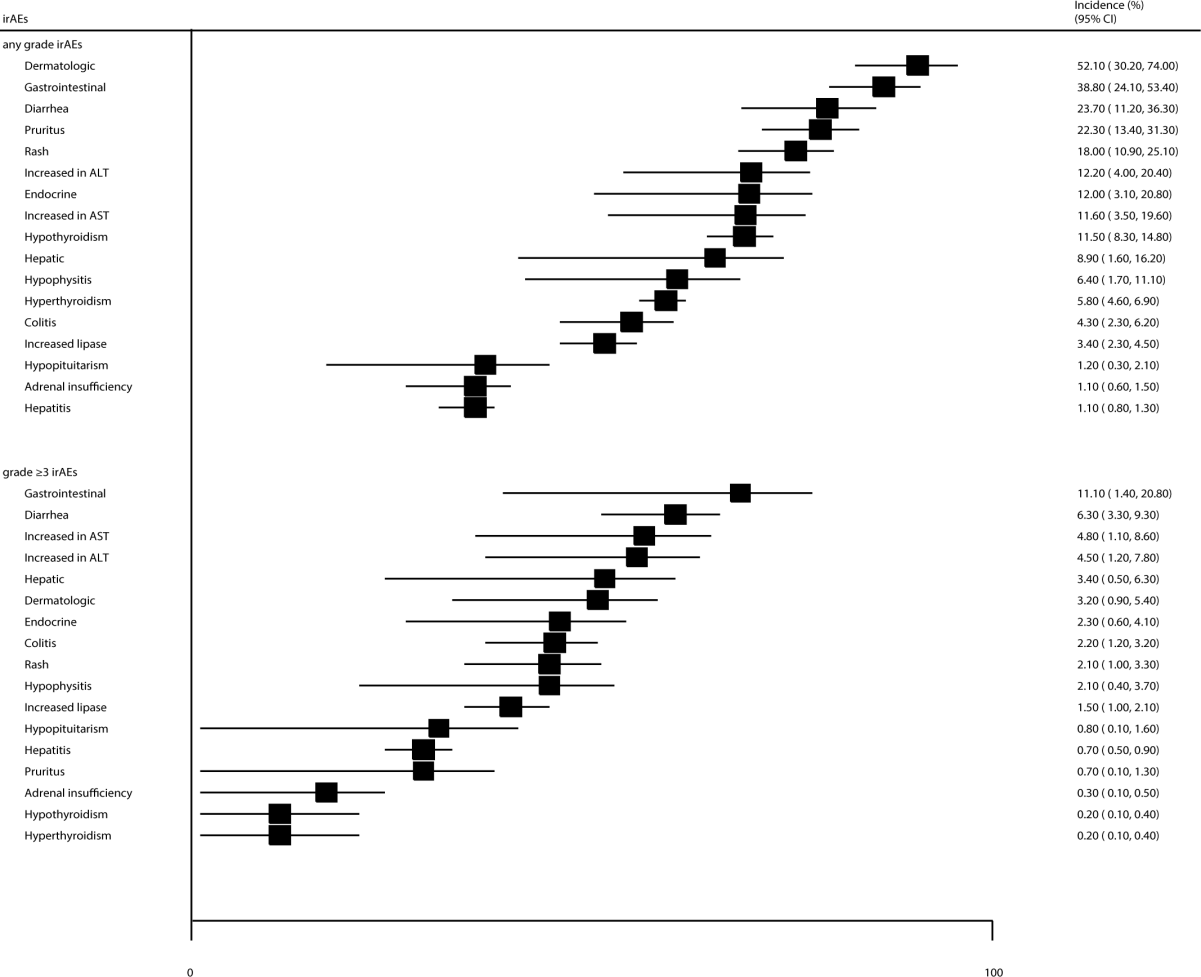
The summary incidences for all-grade and grade ≥ 3 specific irAEs.

The incidences of specific all-grade and grade ≥ 3 irAEs are summarized in **Figure 3**. We noted that the incidences of all-grade dermatologic, gastrointestinal, diarrhea, and pruritus events in patients treated with ICIs were greater than 20%, as follows: 52.1% (95% CI: 30.2–74.0%), 38.8% (95% CI: 24.1–53.4%), 23.7% (95% CI: 11.2–36.3%), and 22.3% (95% CI: 13.4–31.3%), respectively. Moreover, the incidences of specific grade ≥ 3 irAEs for patients treated with ICIs were greater than 3.0%, including gastrointestinal events, diarrhea, increased aspartate aminotransferase and alanine transaminase levels, and hepatic and dermatological event as follows: 11.1% (95% CI: 1.4–20.8%), 6.3% (95% CI: 3.3–9.3%), 4.8% (95% CI: 1.1–8.6%), 4.5% (95% CI: 1.2–7.8%), 3.4% (95% CI: 0.5–6.3%), and 3.2% (95% CI: 0.9–5.4%).

### 3.4 TrAEs

**Figure 4:**
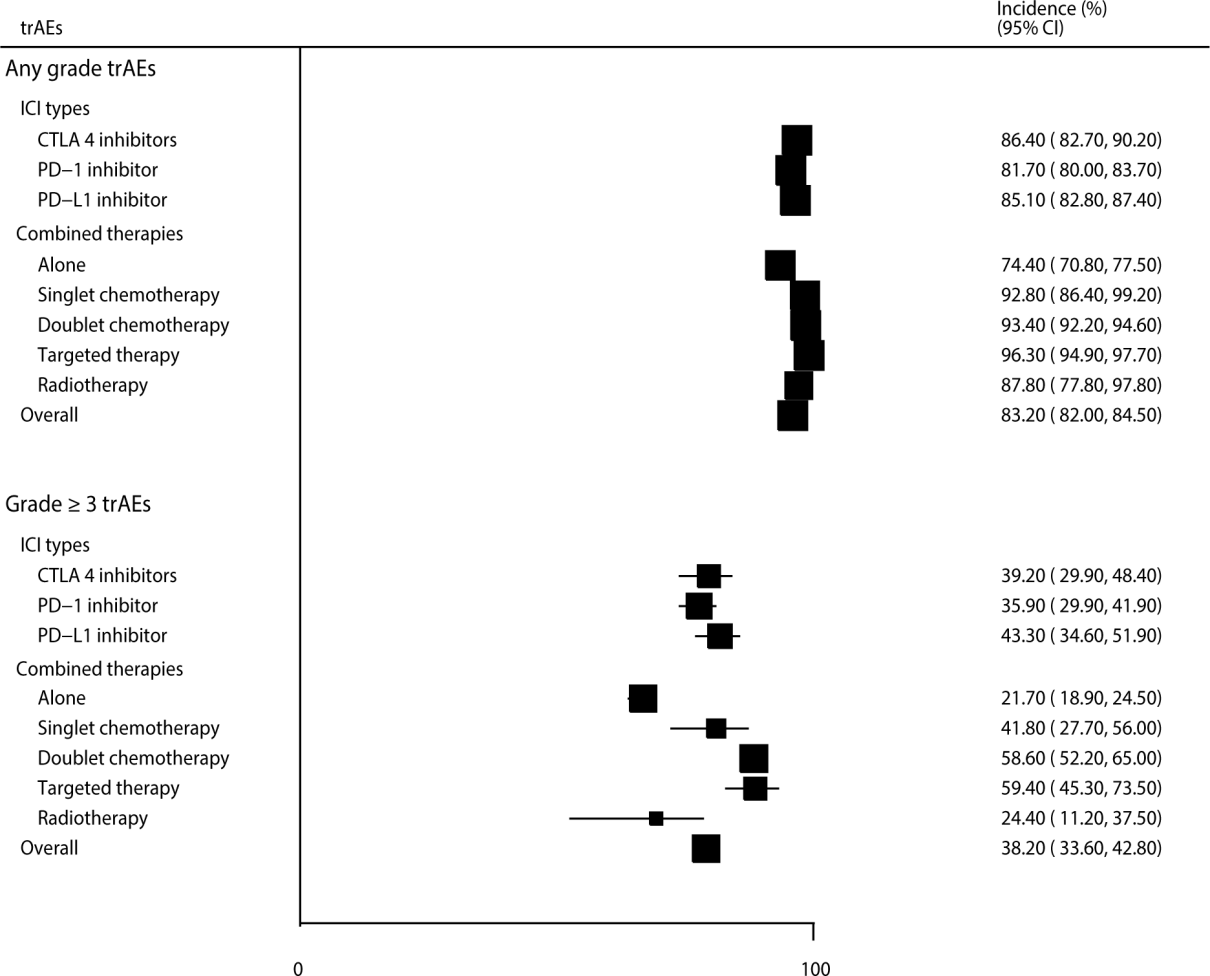
The summary incidences for all-grade and grade ≥ 3 trAEs.

After pooling the included trials, we noted that the incidences of any-grade and grade ≥ 3 trAEs were 83.2% (95% CI: 82.0–84.5%) and 38.2% (95% CI: 33.6–42.8%), respectively. Significant heterogeneity was observed for all-grade (*I^2^ = *98.5%, *P* < 0.001) and grade ≥ 3 trAEs (*I^2^ = *99.3%, *P* < 0.001). When stratified by ICI type, we noted that the incidences of all-grade trAEs for patients treated with CTLA-4, PD-1, and PD-L1 inhibitors were 86.4% (95% CI: 82.7–90.2%), 81.7% (95% CI: 80.0–83.4%), and 85.1% (95% CI: 82.8–87.4%), respectively; for grade ≥ 3 trAEs, these percentages were 39.2% (95% CI: 29.9–48.4%), 35.9% (95% CI: 29.9–41.9%), and 43.3% (95% CI: 34.6–51.9%). When stratified by combined therapy, the incidences of all-grade trAEs for patients treated with ICIs alone, combined with singlet chemotherapy, combined with doublet chemotherapy, combined with targeted therapy, and combined with radiotherapy were 74.4% (95% CI: 70.8–77.5%), 92.8% (95% CI: 86.4–99.2%), 93.4% (95% CI: 92.2–94.6%), 96.3% (95% CI: 94.9–97.7%), and 87.8% (95% CI: 77.8–97.8%), respectively. The respective incidences of grade ≥ 3 trAEs were 21.7% (95% CI: 18.9–24.5%), 41.8% (95% CI: 27.7–56.0%), 58.6% (95% CI: 52.2–65.0%), 59.4% (95% CI: 45.3–73.5%), and 24.4% (95% CI: 11.2–37.5%) (**Figures 4**, **Supplementary 4**).

### 3.5 Specific trAEs

**Figure 5:**
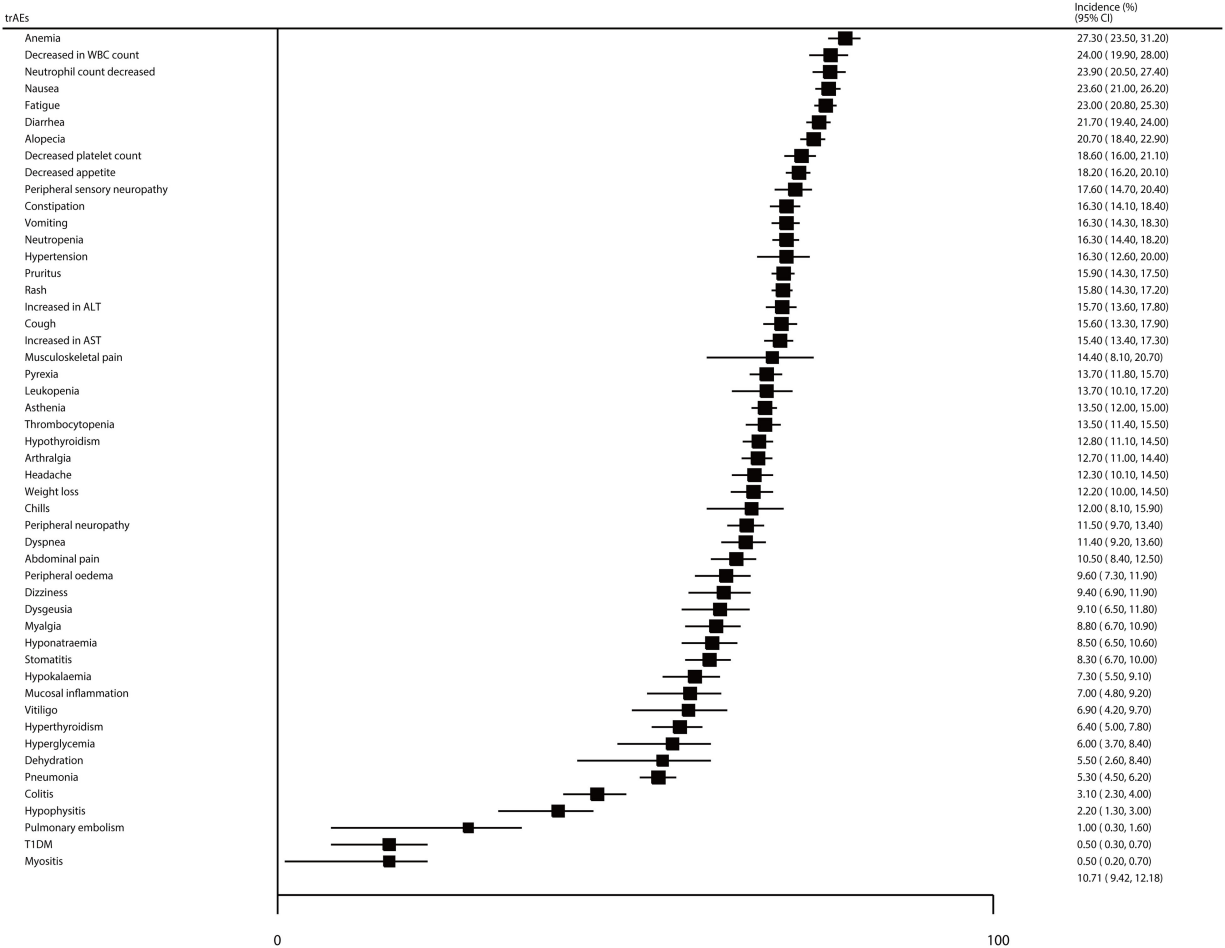
The summary incidences for all-grade specific AEs.

**Figure 6:**
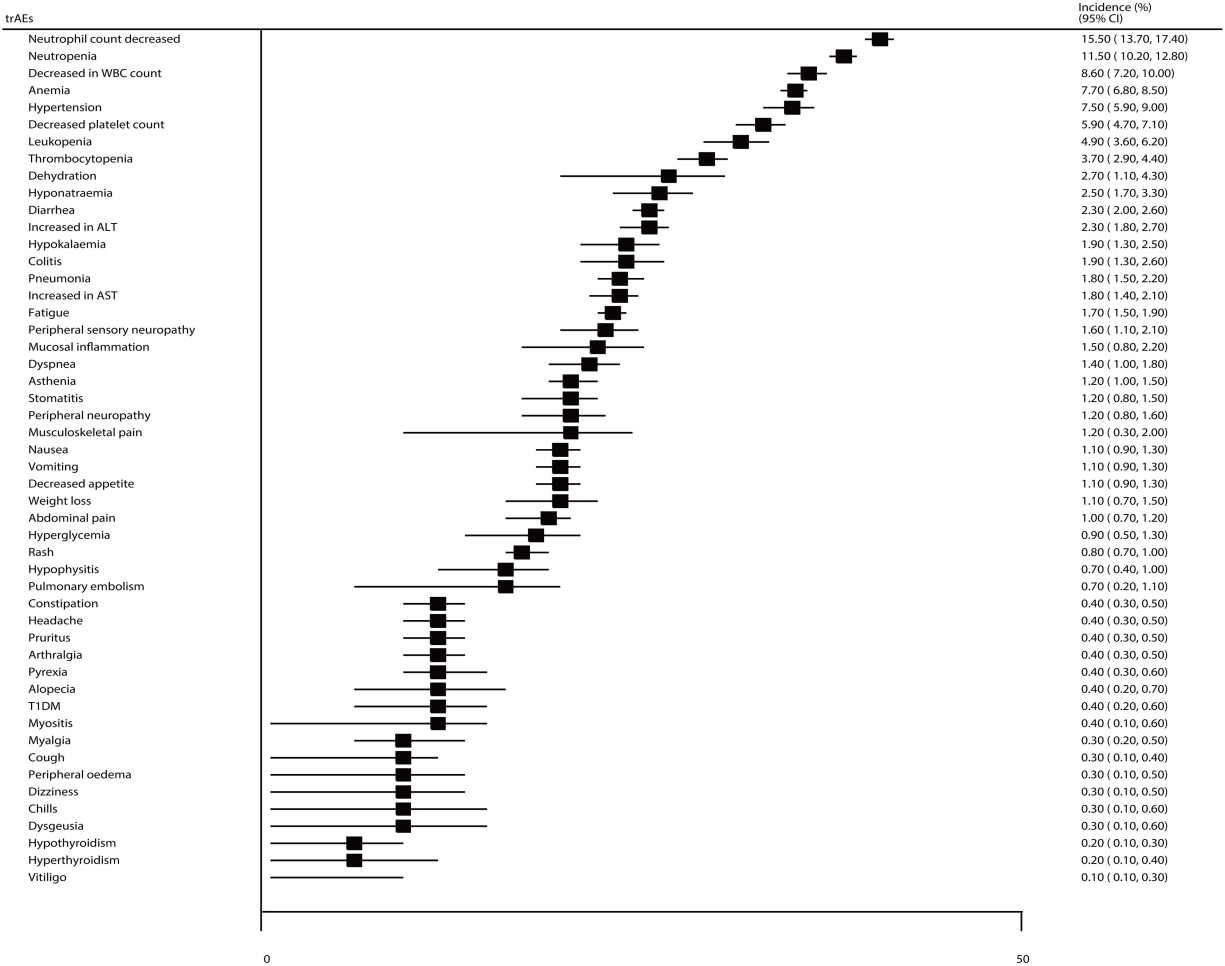
The summary incidences for grade ≥ 3 specific trAEs.

The incidences of specific all-grade trAEs are summarized in **Figure 5**. We noted that the incidences of anemia, decreased WBC count, decreased neutrophil count, nausea, fatigue, diarrhea, and alopecia for patients treated with ICIs were greater than 20%, as follows: 27.3% (95% CI: 23.5–31.2%), 24.0% (95% CI: 19.9–28.0%), 23.9% (95% CI: 20.5–27.4%), 23.6% (95% CI: 21.0–26.2%), 23.0% (95% CI: 20.8–25.3%), 21.7% (95% CI: 19.4–24.0%), and 20.7% (95% CI: 18.4–22.9%), respectively. Moreover, the incidences of specific grade ≥ 3 trAEs, including decreased neutrophil count, neutropenia, decreased WBC count, anemia, hypertension, and decreased platelet count were greater than 5%, as follows: 15.5% (95% CI: 13.7–17.4%), 11.5% (95% CI: 10.2–12.8%), 8.6% (95% CI: 7.2–10.0%), 7.7% (95% CI: 6.8–8.5%), 7.5% (95% CI: 5.9–9.0%), and 5.9% (95% CI: 4.7–7.1%), respectively (**Figure 6**).

### 3.6 Publication bias

There were significant publication biases for grade ≥ 3 irAEs and all-grade or grade ≥ 3 trAEs (**Supplementary 5**), and the conclusions were stabilized for all-grade trAEs and reduced for grade ≥ 3 irAEs and trAEs after adjusting the potential publication bias using the trim and fill method (27).

## 4 Discussion

This comprehensive, quantitative, systematic review and meta-analysis was based on 147 RCTs involving 45,855 patients with cancer at various sites who were randomly treated with 14 different ICIs. The present study is comprehensive as 14 different ICIs as well as all-grade and grade ≥ 3 irAEs and trAEs were included. After reviewing current published trials, we noted that ICIs were always combined with chemotherapy or targeted therapies, and more than half of the patients reported at least one AE. Grade ≥ 3 irAEs and trAEs were not rare, especially for patients receiving CTLA-4 inhibitors or combined targeted therapies. Moreover, the most common all-grade and grade ≥ 3 irAEs and trAEs should be monitored carefully to balance the benefits and adverse effects of ICI therapies.

Several systematic reviews have illustrated the safety profiles of ICIs for cancer treatment at various sites (17, 22, 28–32). Zhou et al. (22) comprehensively assessed the incidences and safety profiles of trAEs among various combination therapies based on 161 RCTs and found that all-grade and grade ≥ 3 trAEs were higher for patients receiving PD-1 or PD-L1 inhibitors combined with chemotherapy or targeted therapies. Inno et al. (28) identified 49 studies and found that the incidence of all-grade and grade 3–4 AEs was 52.2% and 21.5%, respectively, in patients treated with ICIs. Dolladille et al. (29) identified 63 RCTs and reported that ICI use was associated with myocarditis, pericardial disease, heart failure, dyslipidemia, myocardial infarction, and cerebral arterial ischemia. Gu et al. (30) identified 14 RCTs to assess the comprehensive safety profiles of ICIs in patients with advanced non-small cell lung cancer and showed that pembrolizumab caused severe dermatologic irAEs and colitis, nivolumab caused severe endocrine irAEs, and atezolizumab caused severe pneumonitis when combined with platinum-based chemotherapy. Xu et al. (17) investigated the safety profiles of ICIs for esophageal cancer and found that most AEs of combined therapies were tolerable, and all-grade pneumonitis differed between the PD-1 and PD-L1 inhibitor groups. Mei et al. (31) identified 33 RCTs and found that camrelizumab or avelumab combined with chemotherapy showed higher incidences of all-grade AEs, whereas durvalumab and sintilimab could be considered relatively safe PD-L1 and PD-1 inhibitors. Longo et al. (16) identified seven RCTs and found that ICI-based combined treatment was associated with a high risk of grade 3–5 trAEs in patients with small cell lung cancer. Hao et al. (32) showed that ICIs + nab-paclitaxel/paclitaxel were associated with a lower risk of irAEs than that seen with ICI monotherapy. However, previous systematic reviews focused on the safety profiles of specific types of ICIs or in patients with specific cancers. Thus, the current study was performed to extend previous systematic reviews and comprehensively illustrate the safety profiles of ICIs in patients with cancer at various sites.

Our study found that the incidence of all-grade irAEs was higher in patients treated with CTLA-4 inhibitors or ICIs combined with singlet chemotherapy. The reason for the higher risk of irAEs in patients receiving CTLA-4 inhibitors could explained by T cell development at an earlier stage was blocked by CTLA-4 that could directly disrupt central tolerance (33). However, the high risk of irAEs related to the combination of ICIs and singlet chemotherapy might be because only one trial has reported such an outcome; this trial specifically reported 77.7% of any-grade irAEs and 41.7% of grade ≥ 3 irAEs (34). This apparent increase in the AE incidence related to ipilimumab could explain its combination with dacarbazine, which was associated with an increased risk of hepatotoxic events (35, 36). After removing this specific trial, we noted that the incidence of irAEs did not increase rapidly when combined with other antiangiogenic agents. Furthermore, we noticed decreased incidences of irAEs with PD-1 inhibitors compared to that seen with PD-L1 inhibitors, which was not consistent with the findings of previous meta-analyses (32). The binding of PD-1 to both PD-L1 and PD-L2 could be blocked by PD-1 antibody, presenting more comprehensive inhibition of the immune escape pathway (37). The combination regimens were also found to affect the incidence of irAEs, and further meta-analysis should be performed to compare the risk of irAEs between PD-1 and PD-L1 inhibitors. Moreover, the most common irAEs related to ICIs were dermatological and gastrointestinal, whereas the most severe ones were gastrointestinal and endocrine disorders, which should be carefully monitored in clinical practice. Finally, although the incidence of colitis was low, most cases were severe.

Similarly, the incidences of all-grade and grade ≥ 3 trAEs were higher for patients who received ICIs, and the most common trAEs were hematologic toxicity, including anemia, decreased WBC count, and decreased neutrophil count. As expected, these hematological toxicities could be explained by the use of ICIs combined with chemotherapy or radiotherapy (38). Moreover, combined treatments could explain the higher incidence of all-grade alopecia; most trAEs were tolerable, and only 0.4% of patients reported grade ≥ 3 alopecia. Several trAEs related to ICIs are noteworthy, including hypertension, hematological and gastrointestinal disorders, especially those associated with the concomitant use of CTLA-4 inhibitors or targeted therapies (33).

This study had several limitations. First, the incidence of irAEs and trAEs was obtained based on MeDRA in individual trials, whereas some cases presented overlapping MeDRA definitions. Second, there was significant heterogeneity in irAEs, trAEs, and mostly specific AEs, which were not fully explained by stratified analyses based on ICI types and combined therapies. Third, the differences between various ICIs and combined therapies were compared indirectly, and further direct comparisons of results should be explored in large-scale real-world studies. Fourth, the combination treatments and cancer sites differed among included trials, which could affect the incidence of irAEs and trAEs. Further study should address the combination treatments for patients with specific cancer. Finally, the inevitable publication bias restricted the detailed meta-analysis of published data.

## 5 Conclusions

Our study systematically summarized the safety profiles of irAEs and trAEs associated with ICIs in patients with cancer at various sites. We noted that CTLA-4 inhibitors showed a higher risk of irAEs and trAEs than PD-1 or PD-L1 inhibitors. Moreover, the combination of ICIs and targeted therapies presented a higher risk of trAEs, whereas the risk of irAEs was not affected by combined therapies. The results of this study provide a clinical reference to balance the benefits and harms of ICIs treatment.

## Data availability statement

The original contributions presented in the study are included in the article/**Supplementary Material**. Further inquiries can be directed to the corresponding authors.

## Author contributions

XS: Conceptualization, Formal Analysis, Investigation, Writing – original draft. JY: Conceptualization, Formal Analysis, Investigation, Writing – original draft. GQ: Data curation, Writing – review & editing. MS: Data curation, Writing – review & editing. YW: Data curation, Writing – review & editing. GL: Data curation, Writing – review & editing. JQY: Data curation, Writing – review & editing.
