# Supplementary material for: Treatment-related adverse events of immune checkpoint inhibitors in clinical trials: a systematic review and meta-analysis
Source: Front Oncol. 2024 May 17;14:1391724. doi: 10.3389/fonc.2024.1391724 (PMC11140092; doi:10.3389/fonc.2024.1391724)
Supplement: Supplementary file 1 [file DataSheet_1.pdf]

Supplemental file 1

Search strategy in PubMed:

((Immune Checkpoint Inhibitors[MeSH Terms]) OR (Checkpoint Inhibitors, Immune[Title/Abstract])) OR (Immune Checkpoint Inhibitor[Title/Abstract])) OR (Checkpoint Inhibitor, Immune[Title/Abstract])) OR (Immune Checkpoint Blockers[Title/Abstract])) OR (Checkpoint Blockers, Immune[Title/Abstract])) OR (Immune Checkpoint Blockade[Title/Abstract])) OR (Checkpoint Blockade, Immune[Title/Abstract])) OR (Immune Checkpoint Inhibition[Title/Abstract])) OR (Checkpoint Inhibition, Immune[Title/Abstract])) OR (PD-L1 Inhibitors[Title/Abstract])) OR (PD L1 Inhibitors[Title/Abstract])) OR (PD-L1 Inhibitor[Title/Abstract])) OR (PD L1 Inhibitor[Title/Abstract])) OR (Programmed Death-Ligand 1 Inhibitors[Title/Abstract])) OR (Programmed Death Ligand 1 Inhibitors[Title/Abstract])) OR (PD-1-PD-L1 Blockade[Title/Abstract])) OR (Blockade, PD-1-PD-L1[Title/Abstract])) OR (PD 1 PD L1 Blockade[Title/Abstract])) OR (CTLA-4 Inhibitors[Title/Abstract])) OR (CTLA 4 Inhibitors[Title/Abstract])) OR (CTLA-4 Inhibitor[Title/Abstract])) OR (CTLA 4 Inhibitor[Title/Abstract])) OR (Cytotoxic T-Lymphocyte-Associated Protein 4 Inhibitors[Title/Abstract])) OR (Cytotoxic T Lymphocyte Associated Protein 4 Inhibitors[Title/Abstract])) OR (Cytotoxic T-Lymphocyte-Associated Protein 4 Inhibitor[Title/Abstract])) OR (Cytotoxic T Lymphocyte Associated Protein 4 Inhibitor[Title/Abstract])) OR (PD-1 Inhibitors[Title/Abstract])) OR (PD 1 Inhibitors[Title/Abstract])) OR (PD-1 Inhibitor[Title/Abstract])) OR (Inhibitor, PD-1[Title/Abstract])) OR (PD 1 Inhibitor[Title/Abstract])) OR (Programmed Cell Death Protein 1 Inhibitor[Title/Abstract])) OR (Programmed Cell Death Protein 1 Inhibitors[Title/Abstract])) OR (nivolumab[Title/Abstract])) OR (pembrolizumab[Title/Abstract])) OR (atezolizumab[Title/Abstract])) OR (ipilimumab[Title/Abstract])) OR (camrelizumab[Title/Abstract])) OR (toripalimab[Title/Abstract])) OR (tislelizumab[Title/Abstract])) OR (sintilimab[Title/Abstract])) OR (durvalumab[Title/Abstract])) OR (envafolimab[Title/Abstract])) OR (cemiplimab[Title/Abstract]).

Supplemental file 2. The summarized characteristics of included trials and involved patients

Study	Register No.	Country	Sample size	Age (years)	Male (%)	Cancer type	Intervention	Background therapies
Hodi 2010 (1)	NCT00094653	Multinational	540	55.9	60.7	Melanoma	Ipilimumab	No
Robert 2011 (2)	NCT00324155	Multinational	250	57.5	60.8	Melanoma	Ipilimumab	Singlet chemotherapy
Reck 2013 (3)	NCT00527735	Multinational	43	57.0	77.0	SCLC	Ipilimumab	Doublet chemotherapy
Eggermont 2015 (4)	NCT00636168	Multinational	475	51.0	62.0	Melanoma	Ipilimumab	No
Kwon 2014 (5)	NCT00861614	Multinational	399	69.0	100.0	PC	Ipilimumab	Radiotherapy
Nanda 2020 (6)	NCT01042379	Multinational	69	50.0	0.0	BC	Pembrolizumab	Doublet chemotherapy
Beer 2017 (7)	NCT01057810	Multinational	400	70.0	100.0	PC	Ipilimumab	No
Govindan 2017 (8)	NCT01285609	Multinational	388	64.0	84.0	NSCLC	Ipilimumab	Doublet chemotherapy
Reck 2016 (9)	NCT01450761	Multinational	478	62.0	66.0	SCLC	Ipilimumab	Doublet chemotherapy
Bang 2017 (10)	NCT01585987	Multinational	57	65.0	63.2	GC, GEJC	Ipilimumab	Doublet chemotherapy
Brahmer 2015 (11)	NCT01642004	Multinational	135	62.0	82.0	NSCLC	Nivolumab	No
Motzer 2015 (12)	NCT01668784	Multinational	410	62.0	77.0	RCC	Nivolumab	No
Borghaei 2015 (13)	NCT01673867	Multinational	292	61.0	52.0	NSCLC	Nivolumab	Doublet chemotherapy
Ribas 2015 (14)	NCT01704287	Multinational	361	61.0	59.0	Melanoma	Pembrolizumab	No
Weber 2015 (15)	NCT01721746	Multinational	272	59.0	65.0	Melanoma	Nivolumab	No
Robert 2015 (16)	NCT01721772	Multinational	210	64.0	57.6	Melanoma	Nivolumab	No
Maio 2017 (17)	NCT01843374	Multinational	382	66.0	74.0	Mesothelioma	Tremelimumab	No
Larkin 2015 (18)	NCT01844505	Multinational	631	60.0	64.0	Melanoma	Nivolumab, Ipilimumab	No
Robert 2015 (19)	NCT01866319	Multinational	834	62.0	59.6	Melanoma	Pembrolizumab, Ipilimumab	No
Diefenbach 2020 (20)	NCT01896999	Multinational	39	36.2	51.3	HL	Nivolumab, Ipilimumab	Targeted therapy
Fehrenbacher 2016 (21)	NCT01903993	Multinational	144	62.0	65.0	NSCLC	Atezolizumab	No
Herbst 2016 (22)	NCT01905657	Multinational	690	63.0	62.0	NSCLC	Pembrolizumab	No
Postow 2015 (23)	NCT01927419	Multinational	47	67.0	68.0	Melanoma	Ipilimumab	No
Rittmeyer 2017 (24)	NCT02008227	Multinational	425	63.0	61.0	NSCLC	Atezolizumab	No
Reardon 2020 (25)	NCT02017717	Multinational	184	55.5	63.0	Glioblastoma	Nivolumab	No
Langer 2016 (26)	NCT02039674	Multinational	60	62.5	37.0	NSCLC	Pembrolizumab	Doublet chemotherapy
Carbone 2017 (27)	NCT02041533	Multinational	271	63.0	68.0	NSCLC	Nivolumab	No
Ferris 2016 (28)	NCT02105636	Multinational	240	59.0	82.1	HNC	Nivolumab	No
Antonia 2017 (29)	NCT02125461	Multinational	476	64.0	70.2	NSCLC	Durvalumab	No
Reck 2016 (30)	NCT02142738	Multinational	154	64.5	59.7	NSCLC	Pembrolizumab	No
Mok 2019 (31)	NCT02220894	Multinational	637	63.0	70.0	NSCLC	Pembrolizumab	No
Cohen 2019 (32)	NCT02252042	Multinational	247	60.0	84.0	HNC	Pembrolizumab	Doublet chemotherapy
Bellmunt 2017 (33)	NCT02256436	Multinational	270	67.0	74.1	UC	Pembrolizumab	Doublet chemotherapy
Kang 2017 (34)	NCT02267343	Multinational	330	62.0	69.0	GC, GEJC	Nivolumab	No
Powles 2018 (35)	NCT02302807	Multinational	467	67.0	78.0	UC	Atezolizumab	No
Siu 2019 (36)	NCT02319044	Multinational	134	61.5	79.9	HNC	Durvalumab, Tremelimumab	No

Planchard 2020 (37)	NCT02352948	Multinational	177	63.2	63.3	NSCLC	Durvalumab, Tremelimumab	No
Burtness 2019 (38)	NCT02358031	Multinational	582	61.5	81.4	HNC	Pembrolizumab	Doublet chemotherapy
Eggermont 2018 (39)	NCT02362594	Multinational	514	54.0	63.0	Melanoma	Pembrolizumab	No
Socinski 2018 (40)	NCT02366143	Multinational	400	63.0	60.0	NSCLC	Atezolizumab	Doublet chemotherapy
West 2019 (41)	NCT02367781	Multinational	483	64.0	57.0	NSCLC	Atezolizumab	Doublet chemotherapy
Jotte 2020 (42)	NCT02367794	Multinational	666	65.5	83.8	NSCLC	Atezolizumab	Doublet chemotherapy
Ferris 2020 (43)	NCT02369874	Multinational	240	59.0	84.2	HNC	Durvalumab	No
Shitara 2018 (44)	NCT02370498	Multinational	296	62.5	68.0	GC, GEJC	Pembrolizumab	No
Long 2018 (45)	NCT02374242	Australia	41	58.3	73.2	Melanoma	Nivolumab	No
Weber 2017 (46)	NCT02388906	Multinational	906	55.0	58.2	Melanoma	Nivolumab, Ipilimumab	No
Barlesi 2018 (47)	NCT02395172	Multinational	396	64.0	68.0	NSCLC	Avelumab	No
Herbst 2020 (48)	NCT02409342	Multinational	277	64.0	70.8	NSCLC	Atezolizumab	No
Rini 2019 (49)	NCT02420821	Multinational	454	62.0	70.0	RCC	Atezolizumab	Targeted therapy
Schmid 2020 (50)	NCT02425894	Multinational	451	55.0	1.0	BC	Atezolizumab	Singlet chemotherapy
Bellmunt 2021 (51)	NCT02450331	Multinational	406	67.0	79.0	UC	Atezolizumab	No
Rizvi 2020 (52)	NCT02453282	Multinational	163	64.0	69.3	NSCLC	Durvalumab	No
Yang 2019 (53)	NCT02454933	Multinational	12	56.0	50.0	NSCLC	Durvalumab	Targeted therapy
Hellmann 2018 (54)	NCT02477826	Multinational	396	64.0	69.0	NSCLC	Nivolumab	No
Shitara 2020 (55)	NCT02494583	Multinational	513	61.5	73.1	GC	Pembrolizumab	Doublet chemotherapy
O’Brien 2022 (56)	NCT02504372	Multinational	590	65.0	68.0	NSCLC	Pembrolizumab	No
Kelley 2021 (57)	NCT02519348	Multinational	173	63.5	86.1	HCC	Durvalumab, Tremelimumab	No
Park 2022 (58)	NCT02520453	Korea	45	64.0	96.0	EC	Durvalumab	No
Zimmer 2020 (59)	NCT02523313	Germany	59	57.0	53.0	Melanoma	Nivolumab	No
Winer 2021 (60)	NCT02555657	Multinational	312	50.0	0.0	BC	Pembrolizumab	No
Andre 2020 (61)	NCT02563002	Multinational	153	63.0	46.0	CRC	Pembrolizumab	No
Kojima 2020 (62)	NCT02564263	Multinational	314	63.0	86.9	EC	Pembrolizumab	No
Kato 2019 (63)	NCT02569242	Multinational	210	64.0	85.0	EC	Nivolumab	No
Yau 2022 (64)	NCT02576509	Multinational	371	65.0	85.0	HCC	Nivolumab	No
Mateos 2019 (65)	NCT02576977	Multinational	125	65.0	62.0	Myeloma	Pembrolizumab	Immunotherapy
Gandhi 2018 (66)	NCT02578680	Multinational	410	65.0	62.0	NSCLC	Pembrolizumab	Doublet chemotherapy
Usmani 2019 (67)	NCT02579863	Multinational	151	74.0	46.0	Myeloma	Pembrolizumab	Immunotherapy
Pujade-Lauraine 2021 (68)	NCT02580058	Multinational	376	60.5	0.0	OC	Avelumab	Singlet chemotherapy
Chan 2023 (69)	NCT02611960	Multinational	117	51.0	83.8	NPC	Pembrolizumab	No
Wu 2019 (70)	NCT02613507	Multinational	338	60.0	78.0	NSCLC	Nivolumab	No
Bang 2018 (71)	NCT02625623	Multinational	185	59.0	75.7	GC, GEJC	Avelumab	No
Bajorin 2021 (72)	NCT02632409	Multinational	353	65.3	75.1	UC	Nivolumab	No
Nishio 2021 (73)	NCT02657434	Multinational	292	64.0	65.8	NSCLC	Atezolizumab	Doublet chemotherapy
Motzer 2019 (74)	NCT02684006	Multinational	442	62.0	71.5	RCC	Avelumab	Targeted therapy
Kuruvilla 2021 (75)	NCT02684292	Multinational	151	36.0	56.0	HL	Pembrolizumab	No

Finn 2020 (76)	NCT02702401	Multinational	278	67.0	81.3	HCC	Pembrolizumab	No
Lee 2020 (77)	NCT02715531	Multinational	119	61.5	86.6	HCC	Atezolizumab	Targeted therapy
Scherpereel 2019 (78)	NCT02716272	France	63	71.2	75.0	PM	Nivolumab	No
Monk 2021 (79)	NCT02718417	Multinational	663	59.5	0.0	OC	Avelumab	Doublet chemotherapy
Kelly 2021 (80)	NCT02743494	Multinational	532	62.0	84.0	EC, GEJC	Nivolumab	No
Kang 2022 (81)	NCT02746796	Multinational	362	64.0	70.0	GC, GEJC	Nivolumab	Doublet chemotherapy
Horn 2018 (82)	NCT02763579	Multinational	201	64.0	64.2	SCLC	Atezolizumab	Doublet chemotherapy
Paz-Ares 2018 (83)	NCT02775435	Multinational	278	65.0	79.1	NSCLC	Pembrolizumab	Doublet chemotherapy
Gettinger 2021 (84)	NCT02785952	Multinational	127	68.1	68.0	NSCLC	Nivolumab	No
Eng 2019 (85)	NCT02788279	Multinational	273	57.3	60.8	CRC	Atezolizumab	Targeted therapy
Galsky 2020 (86)	NCT02807636	Multinational	813	68.1	76.0	UC	Atezolizumab	Doublet chemotherapy
Choueiri 2023 (87)	NCT02811861	Multinational	355	64.0	72.0	RCC	Pembrolizumab	Targeted therapy
Cortes 2020 (88)	NCT02819518	Multinational	566	53.0	0.0	BC	Pembrolizumab	Doublet chemotherapy
Rini 2019 (89)	NCT02853331	Multinational	432	62.0	71.3	RCC	Pembrolizumab	Targeted therapy
Shah 2021 (90)	NCT02864381	Multinational	144	61.5	68.5	GC, GEJC	Nivolumab	Immunotherapy
Ren 2023 (91)	NCT02864394	Multinational	213	61.0	73.7	NSCLC	Pembrolizumab	No
Chen 2022 (92)	NCT02866383	Multinational	41	63.0	53.7	PcC	Nivolumab	Radiotherapy
Janjigian 2021 (93)	NCT02872116	Multinational	789	62.0	68.0	GC, EC, GEJC	Nivolumab	Doublet chemotherapy
Gutzmer 2020 (94)	NCT02908672	Multinational	256	54.0	59.0	Melanoma	Atezolizumab	Targeted therapy
Schoenfeld 2020 (95)	NCT02919683	USA	14	64.4	71.4	OCC	Nivolumab	No
Vano 2022 (96)	NCT02960906	France	58	61.8	67.2	RCC	Nivolumab	No
Qin 2020 (97)	NCT02989922	China	217	49.0	90.0	HCC	Camrelizumab	No
Forde 2022 (98)	NCT02998528	Multinational	179	64.0	71.5	NSCLC	Nivolumab	Doublet chemotherapy
Chung 2022 (99)	NCT03019588	Multinational	47	61.0	68.0	GC, GEJC	Pembrolizumab	No
Pal 2022 (100)	NCT03024996	Multinational	390	60.5	74.0	RCC	Atezolizumab	No
Schmid 2022 (101)	NCT03036488	Multinational	784	49.0	0.1	BC	Pembrolizumab	Doublet chemotherapy
Moore 2021 (102)	NCT03038100	Multinational	651	60.0	0.0	OC	Atezolizumab	Doublet chemotherapy
Goldman 2021 (103)	NCT03043872	Multinational	268	62.0	71.0	SCLC	Durvalumab	Doublet chemotherapy
Qin 2023 (104)	NCT03062358	Multinational	300	54.0	85.7	HCC	Pembrolizumab	No
Fennell 2021 (105)	NCT03063450	UK	221	70.0	76.0	Mesothelioma	Nivolumab	No
Weber 2023 (106)	NCT03068455	Multinational	924	55.0	58.1	Melanoma	Nivolumab	No
Sezer 2021 (107)	NCT03088540	Multinational	356	63.0	88.0	NSCLC	Cemiplimab	No
Sahai 2022 (108)	NCT03101566	USA	35	60.4	54.3	Biliary cancer	Nivolumab	Doublet chemotherapy
Sugawara 2021 (109)	NCT03117049	Multinational	275	66.0	74.5	NSCLC	Nivolumab	Doublet chemotherapy and targeted therapy
Zhou 2021 (110)	NCT03134872	China	205	59.0	71.0	NSCLC	Camrelizumab	Doublet chemotherapy
Choueiri 2021 (111)	NCT03141177	Multinational	323	62.0	77.1	RCC	Nivolumab	Targeted therapy
Choueiri 2021 (112)	NCT03142334	Multinational	496	60.0	70.0	RCC	Pembrolizumab	No
Doki 2022 (113)	NCT03143153	Multinational	321	64.0	79.0	EC	Nivolumab	Doublet chemotherapy
Lee 2023 (114)	NCT03191786	Multinational	302	75.0	73.0	NSCLC	Atezolizumab	No
Mittendorf 2020 (115)	NCT03197935	Multinational	165	51.0	0.0	BC	Atezolizumab	Doublet chemotherapy
Kaseb 2022 (116)	NCT03222076	USA	13	64.0	85.0	HCC	Nivolumab	No
Tewari 2022 (117)	NCT03257267	Multinational	304	51.0	0.0	CC	Cemiplimab	No

Boyer 2021 (118)	NCT03302234	Multinational	284	65.0	67.3	NSCLC	Pembrolizumab	No
Zhou 2023 (119)	NCT03358875	Multinational	535	61.0	77.8	NSCLC	Tislelizumab	No
Gogishvili 2022 (120)	NCT03409614	Multinational	312	63.0	85.9	NSCLC	Cemiplimab	Doublet chemotherapy
Shen 2022 (121)	NCT03430843	Multinational	256	62.0	84.8	EC	Tislelizumab	No
Finn 2020 (122)	NCT03434379	Multinational	336	64.0	82.0	HCC	Atezolizumab	Targeted therapy
Rosenberg 2023 (123)	NCT03459846	Multinational	154	75.5	72.1	UC	Durvalumab	Targeted therapy
Tawbi 2022 (124)	NCT03470922	Multinational	359	62.0	57.4	Melanoma	Nivolumab	No
Makker 2022 (125)	NCT03517449	Multinational	411	64.0	0.0	EmC	Pembrolizumab	Targeted therapy
Luke 2022 (126)	NCT03553836	Multinational	487	60.0	62.0	Melanoma	Pembrolizumab	No
Cho 2022 (127)	NCT03563716	Multinational	68	68.0	71.0	NSCLC	Atezolizumab	No
Mai 2021 (128)	NCT03581786	Multinational	146	46.0	85.0	NPC	Toripalimab	Doublet chemotherapy
Yang 2020 (129)	NCT03607539	China	266	61.0	76.7	NSCLC	Sintilimab	Doublet chemotherapy
Zhou 2021 (130)	NCT03629925	China	179	64.0	91.1	NSCLC	Sintilimab	Doublet chemotherapy
Colombo 2021 (131)	NCT03635567	Multinational	308	51.0	0.0	CC	Pembrolizumab	Doublet chemotherapy
Lu 2021 (132)	NCT03663205	China	223	60.0	75.3	NSCLC	Tislelizumab	Doublet chemotherapy
Ren 2022 (133)	NCT03668496	China	193	64.0	93.0	NSCLC	Camrelizumab	Doublet chemotherapy
Luo 2021 (134)	NCT03691090	China	298	62.0	87.2	EC	Camrelizumab	Doublet chemotherapy
Yang 2021 (135)	NCT03707509	China	134	52.0	84.0	NPC	Camrelizumab	Doublet chemotherapy
Wang 2022 (136)	NCT03711305	China	230	62.0	80.0	SCLC	Adebrelimab	Doublet chemotherapy
Lu 2022 (137)	NCT03748134	Multinational	327	63.0	85.0	EC	Sintilimab	Doublet chemotherapy
Xu 2023 (138)	NCT03783442	Multinational	326	64.0	87.0	EC	Tislelizumab	Doublet chemotherapy
Zhou 2022 (139)	NCT03789604	China	320	62.0	79.0	NSCLC	Sugemalimab	Doublet chemotherapy
Lu 2022 (140)	NCT03802240	China	293	58.0	41.0	NSCLC	Sintilimab	Doublet chemotherapy and targeted therapy
Wang 2022 (141)	NCT03829969	China	257	63.0	84.4	EC	Toripalimab	Doublet chemotherapy
Antonarakis 2023 (142)	NCT03834519	Multinational	529	71.0	100.0	PC	Pembrolizumab	Targeted therapy
Eskander 2023 (143)	NCT03914612	Multinational	405	66.3	0.0	EmC	Pembrolizumab	Doublet chemotherapy
Hu 2022 (144)	NCT03926338	China	34	49.0	67.6	CRC	Toripalimab	Targeted therapy
Fu 2023 (145)	NCT03977272	China	55	61.0	76.3	PcC	Sintilimab	Doublet chemotherapy
Kelley 2023 (146)	NCT04003636	Multinational	533	64.0	53.0	Biliary cancer	Pembrolizumab	Doublet chemotherapy
Pal 2023 (147)	NCT04338269	Multinational	263	62.0	78.0	RCC	Atezolizumab	Targeted therapy

*BC: breast cancer; CC: cervical cancer; CRC: colorectal cancer; EC: esophageal carcinoma; EmC: endometrial cancer; GC: gastric cancer; GEJC: gastroesophageal junction cancer; HCC: hepatocellular carcinoma; HL: Hodgkin lymphoma; HNC: head and neck carcinoma; NPC: nasopharyngeal carcinoma; NSCLC: non-small cell lung cancer; OC: ovarian cancer; OCC: oral cavity cancer; PcC: pancreatic cancer; PC:prostate cancer; PM: pleural mesothelioma; RCC: renal cell carcinoma; SCLC: small cell lung cancer; UC: urothelial carcinoma

Supplemental file 3. The methodological quality assessment of included trials

Study	Random sequence generation	Allocation concealment	Blinding of participants and personnel	Blinding of outcome assessment	Incomplete outcome data	Selective reporting	Other bias
Hodi 2010 (1)	Low	Low	Low	Low	Low	Low	Low
Robert 2011 (2)	Low	Low	Low	Low	Low	Low	Low
Reck 2013 (3)	Low	Unclear	Low	Low	Low	Low	Unclear
Eggermont 2015 (4)	Low	Low	Low	Low	Low	Low	Unclear
Kwon 2014 (5)	Low	Low	Low	Low	Low	Low	Low
Nanda 2020 (6)	Low	Unclear	Low	Low	Low	Low	Unclear
Beer 2017 (7)	Low	Low	Low	Low	Low	Low	Unclear
Govindan 2017 (8)	Low	Low	Low	Low	Low	Low	Low
Reck 2016 (9)	Low	Low	Low	Low	Low	Low	Low
Bang 2017 (10)	Low	Unclear	Low	Low	Low	Low	Unclear
Brahmer 2015 (11)	Low	Low	Low	Low	Low	Low	Low
Motzer 2015 (12)	Low	Low	Low	Low	Low	Low	Low
Borghaei 2015 (13)	Low	Low	Low	Low	Low	Low	Low
Ribas 2015 (14)	Low	Unclear	Low	Low	Low	Low	Unclear
Weber 2015 (15)	Low	Unclear	Low	Low	Low	Low	Unclear
Robert 2015 (16)	Low	Low	Low	Low	Low	Low	Low
Maio 2017 (17)	Low	Low	Low	Low	Low	Low	Low
Larkin 2015 (18)	Low	Low	Low	Low	Low	Low	Low
Robert 2015 (19)	Low	Low	Low	Low	Low	Low	Low
Diefenbach 2020 (20)	Low	Unclear	Low	Low	Low	Low	Low
Fehrenbacher 2016 (21)	Low	Unclear	Low	Low	Low	Low	Low
Herbst 2016 (22)	Low	Unclear	Low	Low	Low	Low	Low
Postow 2015 (23)	Low	Unclear	Low	Low	Low	Low	Low
Rittmeyer 2017 (24)	Low	Unclear	Low	Low	Low	Low	Low
Reardon 2020 (25)	Low	Unclear	Low	Low	Low	Low	Low
Langer 2016 (26)	Low	Low	Low	Low	Low	Low	Low
Carbone 2017 (27)	Low	Unclear	Low	Low	Low	Low	Low
Ferris 2016 (28)	Low	Unclear	Low	Low	Low	Low	Low
Antonia 2017 (29)	Low	Low	Low	Low	Low	Low	Low
Reck 2016 (30)	Low	Unclear	Low	Low	Low	Low	Low
Mok 2019 (31)	Low	Unclear	Low	Low	Low	Low	Low
Cohen 2019 (32)	Low	Unclear	Low	Low	Low	Low	Low
Bellmunt 2017 (33)	Low	Unclear	Low	Low	Low	Low	Low
Kang 2017 (34)	Low	Low	Low	Low	Low	Low	Low
Powles 2018 (35)	Low	Unclear	Low	Low	Low	Low	Low
Siu 2019 (36)	Low	Unclear	Low	Low	Low	Low	Unclear
Planchard 2020 (37)	Low	Low	Low	Low	Low	Low	Low
Burtness 2019 (38)	Low	Unclear	Low	Low	Low	Low	Low
Eggermont 2018 (39)	Low	Low	Low	Low	Low	Low	Low

Socinski 2018 (40)	Low	Low	Low	Low	Low	Low	Low
West 2019 (41)	Low	Low	Low	Low	Low	Low	Low
Jotte 2020 (42)	Low	Low	Low	Low	Low	Low	Low
Ferris 2020 (43)	Low	Unclear	Low	Low	Low	Low	Low
Shitara 2018 (44)	Low	Unclear	Low	Low	Low	Low	Low
Long 2018 (45)	Low	Unclear	Low	Low	Low	Low	Low
Weber 2017 (46)	Low	Low	Low	Low	Low	Low	Low
Barlesi 2018 (47)	Low	Unclear	Low	Low	Low	Low	Low
Herbst 2020 (48)	Low	Unclear	Low	Low	Low	Low	Low
Rini 2019 (49)	Low	Unclear	Low	Low	Low	Low	Low
Schmid 2020 (50)	Low	Low	Low	Low	Low	Low	Low
Bellmunt 2021 (51)	Low	Unclear	Low	Low	Low	Low	Unclear
Rizvi 2020 (52)	Low	Unclear	Low	Low	Low	Low	Unclear
Yang 2019 (53)	Low	Unclear	Low	Low	Low	Low	Unclear
Hellmann 2018 (54)	Low	Unclear	Low	Low	Low	Low	Low
Shitara 2020 (55)	Low	Unclear	Low	Low	Low	Low	Low
O'Brien 2022 (56)	Low	Low	Low	Low	Low	Low	Low
Kelley 2021 (57)	Low	Unclear	Low	Low	Low	Low	Unclear
Park 2022 (58)	Low	Low	Low	Low	Low	Low	Unclear
Zimmer 2020 (59)	Low	Low	Low	Low	Low	Low	Low
Winer 2021 (60)	Low	Unclear	Low	Low	Low	Low	Low
Andre 2020 (61)	Low	Unclear	Low	Low	Low	Low	Low
Kojima 2020 (62)	Low	Unclear	Low	Low	Low	Low	Unclear
Kato 2019 (63)	Low	Unclear	Low	Low	Low	Low	Low
Yau 2022 (64)	Low	Low	Low	Low	Low	Low	Low
Mateos 2019 (65)	Low	Low	Low	Low	Low	Low	Low
Gandhi 2018 (66)	Low	Low	Low	Low	Low	Low	Low
Usmani 2019 (67)	Low	Unclear	Low	Low	Low	Low	Low
Pujade-Lauraine 2021 (68)	Low	Unclear	Low	Low	Low	Low	Low
Chan 2023 (69)	Low	Unclear	Low	Low	Low	Low	Low
Wu 2019 (70)	Low	Unclear	Low	Low	Low	Low	Unclear
Bang 2018 (71)	Low	Unclear	Low	Low	Low	Low	Unclear
Bajorin 2021 (72)	Low	Low	Low	Low	Low	Low	Low
Nishio 2021 (73)	Low	Unclear	Low	Low	Low	Low	Low
Motzer 2019 (74)	Low	Low	Low	Low	Low	Low	Low
Kuruvilla 2021 (75)	Low	Low	Low	Low	Low	Low	Low
Finn 2020 (76)	Low	Low	Low	Low	Low	Low	Unclear
Lee 2020 (77)	Low	Unclear	Low	Low	Low	Low	Low
Scherpereel 2019 (78)	Low	Low	Low	Low	Low	Low	Low
Monk 2021 (79)	Low	Unclear	Low	Low	Low	Low	Unclear
Kelly 2021 (80)	Low	Low	Low	Low	Low	Low	Low
Kang 2022 (81)	Low	Low	Low	Low	Low	Low	Low
Horn 2018 (82)	Low	Low	Low	Low	Low	Low	Low

Paz-Ares 2018 (83)	Low	Low	Low	Low	Low	Low	Low
Gettinger 2021 (84)	Low	Unclear	Low	Low	Low	Low	Low
Eng 2019 (85)	Low	Unclear	Low	Low	Low	Low	Low
Galsky 2020 (86)	Low	Unclear	Low	Low	Low	Low	Low
Choueiri 2023 (87)	Low	Low	Low	Low	Low	Low	Low
Cortes 2020 (88)	Low	Low	Low	Low	Low	Low	Low
Rini 2019 (89)	Low	Low	Low	Low	Low	Low	Low
Shah 2021 (90)	Low	Unclear	Low	Low	Low	Low	Unclear
Ren 2023 (91)	Low	Unclear	Low	Low	Low	Low	Unclear
Chen 2022 (92)	Low	Unclear	Low	Low	Low	Low	Low
Janjigian 2021 (93)	Low	Low	Low	Low	Low	Low	Low
Gutzmer 2020 (94)	Low	Low	Low	Low	Low	Low	Low
Schoenfeld 2020 (95)	Low	Unclear	Low	Low	Low	Low	Low
Vano 2022 (96)	Low	Unclear	Low	Low	Low	Low	Low
Qin 2020 (97)	Low	Unclear	Low	Low	Low	Low	Low
Forde 2022 (98)	Low	Unclear	Low	Low	Low	Low	Low
Chung 2022 (99)	Low	Unclear	Low	Low	Low	Low	Unclear
Pal 2022 (100)	Low	Low	Low	Low	Low	Low	Low
Schmid 2022 (101)	Low	Low	Low	Low	Low	Low	Low
Moore 2021 (102)	Low	Low	Low	Low	Low	Low	Unclear
Goldman 2021 (103)	Low	Unclear	Low	Low	Low	Low	Low
Qin 2023 (104)	Low	Low	Low	Low	Low	Low	Unclear
Fennell 2021 (105)	Low	Low	Low	Low	Low	Low	Low
Weber 2023 (106)	Low	Unclear	Low	Low	Low	Low	Low
Sezer 2021 (107)	Low	Unclear	Low	Low	Low	Low	Low
Sahai 2022 (108)	Low	Unclear	Low	Low	Low	Low	Unclear
Sugawara 2021 (109)	Low	Low	Low	Low	Low	Low	Low
Zhou 2021 (110)	Low	Low	Low	Low	Low	Low	Low
Choueiri 2021 (111)	Low	Low	Low	Low	Low	Low	Low
Choueiri 2021 (112)	Low	Low	Low	Low	Low	Low	Low
Doki 2022 (113)	Low	Low	Low	Low	Low	Low	Low
Lee 2023 (114)	Low	Unclear	Low	Low	Low	Low	Low
Mittendorf 2020 (115)	Low	Low	Low	Low	Low	Low	Low
Kaseb 2022 (116)	Low	Unclear	Low	Low	Low	Low	Low
Tewari 2022 (117)	Low	Unclear	Low	Low	Low	Low	Low
Boyer 2021 (118)	Low	Low	Low	Low	Low	Low	Low
Zhou 2023 (119)	Low	Unclear	Low	Low	Low	Low	Unclear
Gogishvili 2022 (120)	Low	Low	Low	Low	Low	Low	Low
Shen 2022 (121)	Low	Unclear	Low	Low	Low	Low	Unclear
Finn 2020 (122)	Low	Unclear	Low	Low	Low	Low	Unclear
Rosenberg 2023 (123)	Low	Unclear	Low	Low	Low	Low	Unclear
Tawbi 2022 (124)	Low	Low	Low	Low	Low	Low	Low
Makker 2022 (125)	Low	Low	Low	Low	Low	Low	Low

Luke 2022 (126)	Low	Low	Low	Low	Low	Low	Low
Cho 2022 (127)	Low	Low	Low	Low	Low	Low	Low
Mai 2021 (128)	Low	Low	Low	Low	Low	Low	Low
Yang 2020 (129)	Low	Low	Low	Low	Low	Low	Unclear
Zhou 2021 (130)	Low	Low	Low	Low	Low	Low	Low
Colombo 2021 (131)	Low	Low	Low	Low	Low	Low	Low
Lu 2021 (132)	Low	Unclear	Low	Low	Low	Low	Unclear
Ren 2022 (133)	Low	Low	Low	Low	Low	Low	Low
Luo 2021 (134)	Low	Low	Low	Low	Low	Low	Low
Yang 2021 (135)	Low	Low	Low	Low	Low	Low	Low
Wang 2022 (136)	Low	Low	Low	Low	Low	Low	Low
Lu 2022 (137)	Low	Low	Low	Low	Low	Low	Low
Xu 2023 (138)	Low	Low	Low	Low	Low	Low	Low
Zhou 2022 (139)	Low	Low	Low	Low	Low	Low	Low
Lu 2022 (140)	Low	Low	Low	Low	Low	Low	Low
Wang 2022 (141)	Low	Low	Low	Low	Low	Low	Low
Antonarakis 2023 (142)	Low	Unclear	Low	Low	Low	Low	Unclear
Eskander 2023 (143)	Low	Low	Low	Low	Low	Low	Low
Hu 2022 (144)	Low	Unclear	Low	Low	Low	Low	Low
Fu 2023 (145)	Low	Unclear	Low	Low	Low	Low	Unclear
Kelley 2023 (146)	Low	Low	Low	Low	Low	Low	Low
Pal 2023 (147)	Low	Low	Low	Low	Low	Low	Low

References

1. Hodi FS, O'Day SJ, McDermott DF, *et al*. Improved survival with ipilimumab in patients with metastatic melanoma. *N Engl J Med*. 2010;363(8):711-23.

2. Robert C, Thomas L, Bondarenko I, *et al*. Ipilimumab plus dacarbazine for previously untreated metastatic melanoma. *N Engl J Med*. 2011;364(26):2517-26.

3. Reck M, Bondarenko I, Luft A, *et al*. Ipilimumab in combination with paclitaxel and carboplatin as first-line therapy in extensive-disease-small-cell lung cancer: results from a randomized, double-blind, multicenter phase 2 trial. *Ann Oncol*. 2013;24(1):75-83.

4. Eggermont AM, Chiarion-Sileni V, Grob JJ, *et al*. Adjuvant ipilimumab versus placebo after complete resection of high-risk stage III melanoma (EORTC 18071): a randomised, double-blind, phase 3 trial. *Lancet Oncol*. 2015;16(5):522-30.

5. Kwon ED, Drake CG, Scher HI, *et al*. Ipilimumab versus placebo after radiotherapy in patients with metastatic castration-resistant prostate cancer that had progressed after docetaxel chemotherapy (CA184-043): a multicentre, randomised, double-blind, phase 3 trial. *Lancet Oncol*. 2014;15(7):700-12.

6. Nanda R, Liu MC, Yau C, *et al*. Effect of Pembrolizumab Plus Neoadjuvant Chemotherapy on Pathologic Complete Response in Women With Early-Stage Breast Cancer: An Analysis of the Ongoing Phase 2 Adaptively Randomized I-SPY2 Trial. *JAMA Oncol.* 2020;6(5):676-684.

7. Beer TM, Kwon ED, Drake CG, *et al*. Randomized, Double-Blind, Phase III Trial of Ipilimumab Versus Placebo in Asymptomatic or Minimally Symptomatic Patients With Metastatic Chemotherapy-Naive Castration-Resistant Prostate Cancer. *J Clin Oncol*. 2017;35(1):40-47.

8. Govindan R, Szczesna A, Ahn MJ, *et al*. Phase III Trial of Ipilimumab Combined With Paclitaxel and Carboplatin in Advanced Squamous Non-Small-Cell Lung Cancer. *J Clin Oncol*. 2017;35(30):3449-3457.

9. Reck M, Luft A, Szczesna A, *et al*. Phase III Randomized Trial of Ipilimumab Plus Etoposide and Platinum Versus Placebo Plus Etoposide and Platinum in Extensive-Stage Small-Cell Lung Cancer. *J Clin Oncol.* 2016;34(31):3740-3748.

10. Bang YJ, Cho JY, Kim YH, *et al*. Efficacy of Sequential Ipilimumab Monotherapy versus Best Supportive Care for Unresectable Locally Advanced/Metastatic Gastric or Gastroesophageal Junction Cancer. *Clin Cancer Res*. 2017;23(19):5671-5678.

11. Brahmer J, Reckamp KL, Baas P, *et al*. Nivolumab versus Docetaxel in Advanced Squamous-Cell Non-Small-Cell Lung Cancer. *N Engl J Med*. 2015;373(2):123-35.

12. Motzer RJ, Escudier B, McDermott DF, *et al*. Nivolumab versus Everolimus in Advanced Renal-Cell Carcinoma. *N Engl J Med*. 2015;373(19):1803-13.

13. Borghaei H, Paz-Ares L, Horn L, *et al*. Nivolumab versus Docetaxel in Advanced Nonsquamous Non-Small-Cell Lung Cancer. *N Engl J Med*. 2015;373(17):1627-39.

14. Ribas A, Puzanov I, Dummer R, *et al*. Pembrolizumab versus investigator-choice chemotherapy for ipilimumab-refractory melanoma (KEYNOTE-002): a randomised, controlled, phase 2 trial. *Lancet Oncol*. 2015;16(8):908-18.

15. Weber JS, D'Angelo SP, Minor D, *et al*. Nivolumab versus chemotherapy in patients with advanced melanoma who progressed after anti-CTLA-4 treatment (CheckMate 037): a randomised, controlled, open-label, phase 3 trial. *Lancet Oncol*. 2015;16(4):375-84.

16. Robert C, Long GV, Brady B, *et al*. Nivolumab in previously untreated melanoma without BRAF mutation. *N Engl J Med*. 2015;372(4):320-30.

17. Maio M, Scherpereel A, Calabrò L, *et al*. Tremelimumab as second-line or third-line treatment in relapsed malignant mesothelioma (DETERMINE): a multicentre, international, randomised, double-blind, placebo-controlled phase 2b trial. *Lancet Oncol*. 2017;18(9): 1261-1273.

18. Larkin J, Chiarion-Sileni V, Gonzalez R, *et al*. Combined Nivolumab and Ipilimumab or Monotherapy in Untreated Melanoma. *N Engl J Med*. 2015;373(1):23-34.

19. Robert C, Schachter J, Long GV, *et al*. Pembrolizumab versus Ipilimumab in Advanced Melanoma. *N Engl J Med*. 2015;372(26):2521-32.

20. Diefenbach CS, Hong F, Ambinder RF, *et al*. Ipilimumab, nivolumab, and brentuximab vedotin combination therapies in patients with relapsed or refractory Hodgkin lymphoma: phase 1 results of an open-label, multicentre, phase 1/2 trial. *Lancet Haematol*. 2020;7(9):e660-e670.

21. Fehrenbacher L, Spira A, Ballinger M, *et al*. Atezolizumab versus docetaxel for patients with previously treated non-small-cell lung cancer (POPLAR): a multicentre, open-label, phase 2 randomised controlled trial. *Lancet*. 2016;387(10030):1837-46.

22. Herbst RS, Baas P, Kim DW, *et al*. Pembrolizumab versus docetaxel for previously treated, PD-L1-positive, advanced non-small-cell lung cancer (KEYNOTE-010): a randomised controlled trial. *Lancet*. 2016;387(10027):1540-1550.

23. Postow MA, Chesney J, Pavlick AC, *et al*. Nivolumab and ipilimumab versus ipilimumab in untreated melanoma. *N Engl J Med*. 2015;372(21):2006-17.

24. Rittmeyer A, Barlesi F, Waterkamp D, *et al*. Atezolizumab versus docetaxel in patients with previously treated non-small-cell lung cancer (OAK): a phase 3, open-label, multicentre randomised controlled trial. *Lancet*. 2017;389(10066):255-265.

25. Reardon DA, Brandes AA, Omuro A, *et al*. Effect of Nivolumab vs Bevacizumab in Patients With Recurrent Glioblastoma: The CheckMate 143 Phase 3 Randomized Clinical Trial. *JAMA Oncol*. 2020;6(7):1003-1010.

26. Langer CJ, Gadgeel SM, Borghaei H, *et al*. Carboplatin and pemetrexed with or without pembrolizumab for advanced, non-squamous non-small-cell lung cancer: a randomised, phase 2 cohort of the open-label KEYNOTE-021 study. *Lancet Oncol.* 2016;17(11):1497-1508.

27. Carbone DP, Reck M, Paz-Ares L, *et al*. First-Line Nivolumab in Stage IV or Recurrent Non-Small-Cell Lung Cancer. *N Engl J Med*. 2017;376(25):2415-2426.

28. Ferris RL, Blumenschein G Jr, Fayette J, *et al*. Nivolumab for Recurrent Squamous-Cell Carcinoma of the Head and Neck. *N Engl J Med*. 2016;375(19):1856-1867.

29. Antonia SJ, Villegas A, Daniel D, *et al*. Durvalumab after Chemoradiotherapy in Stage III Non-Small-Cell Lung Cancer. *N Engl J Med*. 2017 ;377(20):1919-1929.

30. Reck M, Rodríguez-Abreu D, Robinson AG, *et al*. Pembrolizumab versus Chemotherapy for PD-L1-Positive Non-Small-Cell Lung Cancer. *N Engl J Med*. 2016;375(19):1823-1833.

31. Mok TSK, Wu YL, Kudaba I, *et al*. Pembrolizumab versus chemotherapy for previously untreated, PD-L1-expressing, locally advanced or metastatic non-small-cell lung cancer (KEYNOTE-042): a randomised, open-label, controlled, phase 3 trial. *Lancet*. 2019;393(10183): 1819-1830.

32. Cohen EEW, Soulières D, Le Tourneau C, *et al*. Pembrolizumab versus methotrexate, docetaxel, or cetuximab for recurrent or metastatic head-and-neck squamous cell carcinoma (KEYNOTE-040): a randomised, open-label, phase 3 study. *Lancet.* 2019;393(10167):156-167.

33. Bellmunt J, de Wit R, Vaughn DJ, *et al*. Pembrolizumab as Second-Line Therapy for Advanced Urothelial Carcinoma. *N Engl J Med*. 2017;376(11):1015-1026.

34. Kang YK, Boku N, Satoh T, *et al*. Nivolumab in patients with advanced gastric or gastro-oesophageal junction cancer refractory to, or intolerant of, at least two previous

chemotherapy regimens (ONO-4538-12, ATTRACTION-2): a randomised, double-blind, placebo-controlled, phase 3 trial. *Lancet*. 2017;390(10111):2461-2471.

35. Powles T, Durán I, van der Heijden MS, *et al*. Atezolizumab versus chemotherapy in patients with platinum-treated locally advanced or metastatic urothelial carcinoma (IMvigor211): a multicentre, open-label, phase 3 randomised controlled trial. *Lancet*. 2018;391(10122):748-757.

36. Siu LL, Even C, Mesía R, *et al*. Safety and Efficacy of Durvalumab With or Without Tremelimumab in Patients With PD-L1-Low/Negative Recurrent or Metastatic HNSCC: The Phase 2 CONDOR Randomized Clinical Trial. *JAMA Oncol*. 2019;5(2):195-203.

37. Planchard D, Reinmuth N, Orlov S, *et al*. ARCTIC: durvalumab with or without tremelimumab as third-line or later treatment of metastatic non-small-cell lung cancer. *Ann Oncol*. 2020;31(5):609-618.

38. Burtness B, Harrington KJ, Greil R, *et al*. Pembrolizumab alone or with chemotherapy versus cetuximab with chemotherapy for recurrent or metastatic squamous cell carcinoma of the head and neck (KEYNOTE-048): a randomised, open-label, phase 3 study. *Lancet*. 2019;394(10212): 1915-1928.

39. Eggermont AMM, Blank CU, Mandala M, *et al*. Adjuvant Pembrolizumab versus Placebo in Resected Stage III Melanoma. *N Engl J Med*. 2018;378(19):1789-1801.

40. Socinski MA, Jotte RM, Cappuzzo F, *et al*. Atezolizumab for First-Line Treatment of Metastatic Nonsquamous NSCLC. *N Engl J Med*. 2018;378(24):2288-2301.

41. West H, McCleod M, Hussein M, *et al*. Atezolizumab in combination with carboplatin plus nab-paclitaxel chemotherapy compared with chemotherapy alone as first-line treatment for metastatic non-squamous non-small-cell lung cancer (IMpower130): a multicentre, randomised, open-label, phase 3 trial. *Lancet Oncol*. 2019;20(7):924-937.

42. Jotte R, Cappuzzo F, Vynnychenko I, *et al*. Atezolizumab in Combination With Carboplatin and Nab-Paclitaxel in Advanced Squamous NSCLC (IMpower131): Results From a Randomized Phase III Trial. *J Thorac Oncol*. 2020;15(8):1351-1360.

43. Ferris RL, Haddad R, Even C, *et al*. Durvalumab with or without tremelimumab in patients with recurrent or metastatic head and neck squamous cell carcinoma: EAGLE, a randomized, open-label phase III study. *Ann Oncol*. 2020;31(7):942-950.

44. Shitara K, Özgüroğlu M, Bang YJ, *et al*. Pembrolizumab versus paclitaxel for previously

treated, advanced gastric or gastro-oesophageal junction cancer (KEYNOTE-061): a randomised, open-label, controlled, phase 3 trial. *Lancet*. 2018;392(10142):123-133.

45. Long GV, Atkinson V, Lo S, *et al*. Combination nivolumab and ipilimumab or nivolumab alone in melanoma brain metastases: a multicentre randomised phase 2 study. *Lancet Oncol*. 2018; 19(5):672-681.

46. Weber J, Mandala M, Del Vecchio M, *et al*. Adjuvant Nivolumab versus Ipilimumab in Resected Stage III or IV Melanoma. *N Engl J Med.* 2017;377(19):1824-1835.

47. Barlesi F, Vansteenkiste J, Spigel D, *et al*. Avelumab versus docetaxel in patients with platinum-treated advanced non-small-cell lung cancer (JAVELIN Lung 200): an open-label, randomised, phase 3 study. *Lancet Oncol*. 2018;19(11):1468-1479.

48. Herbst RS, Giaccone G, de Marinis F, *et al*. Atezolizumab for First-Line Treatment of PD-L1-Selected Patients with NSCLC. *N Engl J Med*. 2020;383(14):1328-1339.

49. Rini BI, Powles T, Atkins MB, *et al*. Atezolizumab plus bevacizumab versus sunitinib in patients with previously untreated metastatic renal cell carcinoma (IMmotion151): a multicentre, open-label, phase 3, randomised controlled trial. *Lancet*. 2019;393(10189):2404-2415.

50. Schmid P, Rugo HS, Adams S, *et al*. Atezolizumab plus nab-paclitaxel as first-line treatment for unresectable, locally advanced or metastatic triple-negative breast cancer (IMpassion130): updated efficacy results from a randomised, double-blind, placebo-controlled, phase 3 trial. *Lancet Oncol*. 2020;21(1):44-59.

51. Bellmunt J, Hussain M, Gschwend JE, *et al*. Adjuvant atezolizumab versus observation in muscle-invasive urothelial carcinoma (IMvigor010): a multicentre, open-label, randomised, phase 3 trial. *Lancet Oncol*. 2021;22(4):525-537.

52. Rizvi NA, Cho BC, Reinmuth N, *et al*. Durvalumab With or Without Tremelimumab vs Standard Chemotherapy in First-line Treatment of Metastatic Non-Small Cell Lung Cancer: The MYSTIC Phase 3 Randomized Clinical Trial. *JAMA Oncol*. 2020;6(5):661-674.

53. Yang JC, Shepherd FA, Kim DW, *et al*. Osimertinib Plus Durvalumab versus Osimertinib Monotherapy in EGFR T790M-Positive NSCLC following Previous EGFR TKI Therapy: CAURAL Brief Report. *J Thorac Oncol*. 2019;14(5):933-939.

54. Hellmann MD, Ciuleanu TE, Pluzanski A, *et al*. Nivolumab plus Ipilimumab in Lung Cancer with a High Tumor Mutational Burden. *N Engl J Med*. 2018;378(22):2093-2104.

55. Shitara K, Van Cutsem E, Bang YJ, *et al*. Efficacy and Safety of Pembrolizumab or Pembrolizumab Plus Chemotherapy vs Chemotherapy Alone for Patients With First-line, Advanced Gastric Cancer: The KEYNOTE-062 Phase 3 Randomized Clinical Trial. *JAMA Oncol*. 2020;6(10):1571-1580.

56. O'Brien M, Paz-Ares L, Marreaud S, *et al*. Pembrolizumab versus placebo as adjuvant therapy for completely resected stage IB-IIIA non-small-cell lung cancer (PEARLS/KEYNOTE-091): an interim analysis of a randomised, triple-blind, phase 3 trial. *Lancet Oncol*. 2022;23(10): 1274-1286.

57. Kelley RK, Sangro B, Harris W, *et al*. Safety, Efficacy, and Pharmacodynamics of Tremelimumab Plus Durvalumab for Patients With Unresectable Hepatocellular Carcinoma: Randomized Expansion of a Phase I/II Study. *J Clin Oncol*. 2021;39(27):2991-3001.

58. Park S, Sun JM, Choi YL, *et al*. Adjuvant durvalumab for esophageal squamous cell carcinoma after neoadjuvant chemoradiotherapy: a placebo-controlled, randomized, double-blind, phase II study. *ESMO Open*. 2022;7(1):100385.

59. Zimmer L, Livingstone E, Hassel JC, *et al*. Adjuvant nivolumab plus ipilimumab or nivolumab monotherapy versus placebo in patients with resected stage IV melanoma with no evidence of disease (IMMUNED): a randomised, double-blind, placebo-controlled, phase 2 trial. *Lancet*. 2020;395(10236):1558-1568.

60. Winer EP, Lipatov O, Im SA, *et al*. Pembrolizumab versus investigator-choice chemotherapy for metastatic triple-negative breast cancer (KEYNOTE-119): a randomised, open-label, phase 3 trial. *Lancet Oncol*. 2021;22(4):499-511.

61. André T, Shiu KK, Kim TW, *et al*. Pembrolizumab in Microsatellite-Instability-High Advanced Colorectal Cancer. *N Engl J Med*. 2020;383(23):2207-2218.

62. Kojima T, Shah MA, Muro K, *et al*. Randomized Phase III KEYNOTE-181 Study of Pembrolizumab Versus Chemotherapy in Advanced Esophageal Cancer. *J Clin Oncol*. 2020; 38(35):4138-4148.

63. Kato K, Cho BC, Takahashi M, *et al*. Nivolumab versus chemotherapy in patients with advanced oesophageal squamous cell carcinoma refractory or intolerant to previous chemotherapy (ATTRACTION-3): a multicentre, randomised, open-label, phase 3 trial. *Lancet Oncol*. 2019; 20(11):1506-1517.

64. Yau T, Park JW, Finn RS, *et al*. Nivolumab versus sorafenib in advanced hepatocellular carcinoma (CheckMate 459): a randomised, multicentre, open-label, phase 3 trial. *Lancet Oncol*. 2022;23(1):77-90.

65. Mateos MV, Blacklock H, Schjesvold F, *et al*. Pembrolizumab plus pomalidomide and dexamethasone for patients with relapsed or refractory multiple myeloma (KEYNOTE-183): a randomised, open-label, phase 3 trial. *Lancet Haematol.* 2019;6(9):e459-e469.

66. Gandhi L, Rodríguez-Abreu D, Gadgeel S, *et al*. Pembrolizumab plus Chemotherapy in Metastatic Non-Small-Cell Lung Cancer. *N Engl J Med*. 2018;378(22):2078-2092.

67. Usmani SZ, Schjesvold F, Oriol A, *et al*. Pembrolizumab plus lenalidomide and dexamethasone for patients with treatment-naive multiple myeloma (KEYNOTE-185): a randomised, open-label, phase 3 trial. *Lancet Haematol*. 2019;6(9):e448-e458.

68. Pujade-Lauraine E, Fujiwara K, Ledermann JA, *et al*. Avelumab alone or in combination with chemotherapy versus chemotherapy alone in platinum-resistant or platinum-refractory ovarian cancer (JAVELIN Ovarian 200): an open-label, three-arm, randomised, phase 3 study. *Lancet Oncol*. 2021;22(7):1034-1046.

69. Chan ATC, Lee VHF, Hong RL, *et al*. Pembrolizumab monotherapy versus chemotherapy in platinum-pretreated, recurrent or metastatic nasopharyngeal cancer (KEYNOTE-122): an open-label, randomized, phase III trial. *Ann Oncol*. 2023;34(3):251-261.

70. Wu YL, Lu S, Cheng Y, *et al*. Nivolumab Versus Docetaxel in a Predominantly Chinese Patient Population With Previously Treated Advanced NSCLC: CheckMate 078 Randomized Phase III Clinical Trial. *J Thorac Oncol*. 2019;14(5):867-875.

71. Bang YJ, Ruiz EY, Van Cutsem E, *et al*. Phase III, randomised trial of avelumab versus physician's choice of chemotherapy as third-line treatment of patients with advanced gastric or gastro-oesophageal junction cancer: primary analysis of JAVELIN Gastric 300. *Ann Oncol*. 2018; 29(10):2052-2060.

72. Bajorin DF, Witjes JA, Gschwend JE, *et al*. Adjuvant Nivolumab versus Placebo in Muscle-Invasive Urothelial Carcinoma. *N Engl J Med*. 2021;384(22):2102-2114.

73. Nishio M, Barlesi F, West H, *et al*. Atezolizumab Plus Chemotherapy for First-Line Treatment of Nonsquamous NSCLC: Results From the Randomized Phase 3 IMpower132 Trial. *J Thorac Oncol*. 2021;16(4):653-664.

74. Motzer RJ, Penkov K, Haanen J, *et al*. Avelumab plus Axitinib versus Sunitinib for Advanced Renal-Cell Carcinoma. *N Engl J Med*. 2019;380(12):1103-1115.

75. Kuruvilla J, Ramchandren R, Santoro A, *et al*. Pembrolizumab versus brentuximab vedotin in relapsed or refractory classical Hodgkin lymphoma (KEYNOTE-204): an interim analysis of a multicentre, randomised, open-label, phase 3 study. *Lancet Oncol*. 2021;22(4):512-524.

76. Finn RS, Ryoo BY, Merle P, *et al*. Pembrolizumab As Second-Line Therapy in Patients With Advanced Hepatocellular Carcinoma in KEYNOTE-240: A Randomized, Double-Blind, Phase III Trial. *J Clin Oncol*. 2020;38(3):193-202.

77. Lee MS, Ryoo BY, Hsu CH, *et al*. Atezolizumab with or without bevacizumab in unresectable hepatocellular carcinoma (GO30140): an open-label, multicentre, phase 1b study. *Lancet Oncol*. 2020;21(6):808-820.

78. Scherpereel A, Mazieres J, Greillier L, *et al*. Nivolumab or nivolumab plus ipilimumab in patients with relapsed malignant pleural mesothelioma (IFCT-1501 MAPS2): a multicentre, open-label, randomised, non-comparative, phase 2 trial. *Lancet Oncol*. 2019;20(2):239-253.

79. Monk BJ, Colombo N, Oza AM, *et al*. Chemotherapy with or without avelumab followed by avelumab maintenance versus chemotherapy alone in patients with previously untreated epithelial ovarian cancer (JAVELIN Ovarian 100): an open-label, randomised, phase 3 trial. *Lancet Oncol*. 2021;22(9):1275-1289.

80. Kelly RJ, Ajani JA, Kuzdzal J, *et al*. Adjuvant Nivolumab in Resected Esophageal or Gastroesophageal Junction Cancer. *N Engl J Med*. 2021;384(13):1191-1203.

81. Kang YK, Chen LT, Ryu MH, *et al*. Nivolumab plus chemotherapy versus placebo plus chemotherapy in patients with HER2-negative, untreated, unresectable advanced or recurrent gastric or gastro-oesophageal junction cancer (ATTRACTION-4): a randomised, multicentre, double-blind, placebo-controlled, phase 3 trial. *Lancet Oncol*. 2022;23(2):234-247.

82. Horn L, Mansfield AS, Szczęsna A, *et al*. First-Line Atezolizumab plus Chemotherapy in Extensive-Stage Small-Cell Lung Cancer. *N Engl J Med*. 2018;379(23):2220-2229.

83. Paz-Ares L, Luft A, Vicente D, *et al*. Pembrolizumab plus Chemotherapy for Squamous Non-Small-Cell Lung Cancer. *N Engl J Med*. 2018;379(21):2040-2051.

84. Gettinger SN, Redman MW, Bazhenova L, *et al*. Nivolumab Plus Ipilimumab vs Nivolumab for Previously Treated Patients With Stage IV Squamous Cell Lung Cancer: The Lung-MAP

S1400I Phase 3 Randomized Clinical Trial. *JAMA Oncol*. 2021;7(9):1368-1377.

85. Eng C, Kim TW, Bendell J, *et al*. Atezolizumab with or without cobimetinib versus regorafenib in previously treated metastatic colorectal cancer (IMblaze370): a multicentre, open-label, phase 3, randomised, controlled trial. *Lancet Oncol*. 2019;20(6):849-861.

86. Galsky MD, Arija JÁA, Bamias A, *et al*. Atezolizumab with or without chemotherapy in metastatic urothelial cancer (IMvigor130): a multicentre, randomised, placebo-controlled phase 3 trial. *Lancet*. 2020;395(10236):1547-1557.

87. Choueiri TK, Eto M, Motzer R, *et al*. Lenvatinib plus pembrolizumab versus sunitinib as first-line treatment of patients with advanced renal cell carcinoma (CLEAR): extended follow-up from the phase 3, randomised, open-label study. *Lancet Oncol*. 2023;24(3):228-238.

88. Cortes J, Cescon DW, Rugo HS, *et al*. Pembrolizumab plus chemotherapy versus placebo plus chemotherapy for previously untreated locally recurrent inoperable or metastatic triple-negative breast cancer (KEYNOTE-355): a randomised, placebo-controlled, double-blind, phase 3 clinical trial. *Lancet.* 2020;396(10265):1817-1828.

89. Rini BI, Plimack ER, Stus V, *et al*. Pembrolizumab plus Axitinib versus Sunitinib for Advanced Renal-Cell Carcinoma. *N Engl J Med*. 2019;380(12):1116-1127.

90. Shah MA, Cunningham D, Metges JP, *et al*. Randomized, open-label, phase 2 study of andecaliximab plus nivolumab versus nivolumab alone in advanced gastric cancer identifies biomarkers associated with survival. *J Immunother Cancer.* 2021;9(12):e003580.

91. Ren S, Feng J, Ma S, *et al*. KEYNOTE-033: Randomized phase 3 study of pembrolizumab vs docetaxel in previously treated, PD-L1-positive, advanced NSCLC. *Int J Cancer.* 2023;153(3): 623-634.

92. Chen IM, Johansen JS, Theile S, *et al*. Randomized Phase II Study of Nivolumab With or Without Ipilimumab Combined With Stereotactic Body Radiotherapy for Refractory Metastatic Pancreatic Cancer (CheckPAC). *J Clin Oncol*. 2022;40(27):3180-3189.

93. Janjigian YY, Shitara K, Moehler M, *et al*. First-line nivolumab plus chemotherapy versus chemotherapy alone for advanced gastric, gastro-oesophageal junction, and oesophageal adenocarcinoma (CheckMate 649): a randomised, open-label, phase 3 trial. *Lancet.* 2021; 398(10294):27-40.

94. Gutzmer R, Stroyakovskiy D, Gogas H, *et al*. Atezolizumab, vemurafenib, and cobimetinib as

first-line treatment for unresectable advanced BRAFV600 mutation-positive melanoma (IMspire150): primary analysis of the randomised, double-blind, placebo-controlled, phase 3 trial. *Lancet*. 2020;395(10240):1835-1844.

95. Schoenfeld JD, Hanna GJ, Jo VY, *et al*. Neoadjuvant Nivolumab or Nivolumab Plus Ipilimumab in Untreated Oral Cavity Squamous Cell Carcinoma: A Phase 2 Open-Label Randomized Clinical Trial. *JAMA Oncol.* 2020;6(10):1563-1570.

96. Vano YA, Elaidi R, Bennamoun M, *et al*. Nivolumab, nivolumab-ipilimumab, and VEGFR-tyrosine kinase inhibitors as first-line treatment for metastatic clear-cell renal cell carcinoma (BIONIKK): a biomarker-driven, open-label, non-comparative, randomised, phase 2 trial. *Lancet Oncol.* 2022;23(5):612-624.

97. Qin S, Ren Z, Meng Z, *et al*. Camrelizumab in patients with previously treated advanced hepatocellular carcinoma: a multicentre, open-label, parallel-group, randomised, phase 2 trial. *Lancet Oncol.* 2020;21(4):571-580.

98. Forde PM, Spicer J, Lu S, *et al*. Neoadjuvant Nivolumab plus Chemotherapy in Resectable Lung Cancer. *N Engl J Med.* 2022;386(21):1973-1985.

99. Chung HC, Kang YK, Chen Z, *et al*. Pembrolizumab versus paclitaxel for previously treated advanced gastric or gastroesophageal junction cancer (KEYNOTE-063): A randomized, open-label, phase 3 trial in Asian patients. *Cancer.* 2022;128(5):995-1003.

100. Pal SK, Uzzo R, Karam JA, *et al*. Adjuvant atezolizumab versus placebo for patients with renal cell carcinoma at increased risk of recurrence following resection (IMmotion010): a multicentre, randomised, double-blind, phase 3 trial. *Lancet.* 2022;400(10358):1103-1116.

101. Schmid P, Cortes J, Dent R, *et al*. Event-free Survival with Pembrolizumab in Early Triple-Negative Breast Cancer. *N Engl J Med*. 2022;386(6):556-567.

102. Moore KN, Bookman M, Sehouli J, *et al*. Atezolizumab, Bevacizumab, and Chemotherapy for Newly Diagnosed Stage III or IV Ovarian Cancer: Placebo-Controlled Randomized Phase III Trial (IMagyn050/GOG 3015/ENGOT-OV39). *J Clin Oncol.* 2021;39(17):1842-1855.

103. Goldman JW, Dvorkin M, Chen Y, *et al*. Durvalumab, with or without tremelimumab, plus platinum-etoposide versus platinum-etoposide alone in first-line treatment of extensive-stage small-cell lung cancer (CASPIAN): updated results from a randomised, controlled, open-label, phase 3 trial. *Lancet Oncol.* 2021;22(1):51-65.

104. Qin S, Chen Z, Fang W, *et al*. Pembrolizumab Versus Placebo as Second-Line Therapy in Patients From Asia With Advanced Hepatocellular Carcinoma: A Randomized, Double-Blind, Phase III Trial. *J Clin Oncol*. 2023;41(7):1434-1443.

105. Fennell DA, Ewings S, Ottensmeier C, *et al*. Nivolumab versus placebo in patients with relapsed malignant mesothelioma (CONFIRM): a multicentre, double-blind, randomised, phase 3 trial. *Lancet Oncol*. 2021;22(11):1530-1540.

106. Weber JS, Schadendorf D, Del Vecchio M, *et al*. Adjuvant Therapy of Nivolumab Combined With Ipilimumab Versus Nivolumab Alone in Patients With Resected Stage IIIB-D or Stage IV Melanoma (CheckMate 915). *J Clin Oncol*. 2023;41(3):517-527.

107. Sezer A, Kilickap S, Gümüş M, *et al*. Cemiplimab monotherapy for first-line treatment of advanced non-small-cell lung cancer with PD-L1 of at least 50%: a multicentre, open-label, global, phase 3, randomised, controlled trial. *Lancet.* 2021;397(10274):592-604.

108. Sahai V, Griffith KA, Beg MS, *et al*. A randomized phase 2 trial of nivolumab, gemcitabine, and cisplatin or nivolumab and ipilimumab in previously untreated advanced biliary cancer: BilT-01. *Cancer.* 2022;128(19):3523-3530.

109. Sugawara S, Lee JS, Kang JH, *et al*. Nivolumab with carboplatin, paclitaxel, and bevacizumab for first-line treatment of advanced nonsquamous non-small-cell lung cancer. *Ann Oncol.* 2021;32(9):1137-1147.

110. Zhou C, Chen G, Huang Y, *et al*. Camrelizumab plus carboplatin and pemetrexed versus chemotherapy alone in chemotherapy-naive patients with advanced non-squamous non-small-cell lung cancer (CameL): a randomised, open-label, multicentre, phase 3 trial. *Lancet Respir Med.* 2021;9(3):305-314.

111. Choueiri TK, Powles T, Burotto M, *et al*. Nivolumab plus Cabozantinib versus Sunitinib for Advanced Renal-Cell Carcinoma. *N Engl J Med.* 2021;384(9):829-841.

112. Choueiri TK, Tomczak P, Park SH, *et al*. Adjuvant Pembrolizumab after Nephrectomy in Renal-Cell Carcinoma. *N Engl J Med.* 2021;385(8):683-694.

113. Doki Y, Ajani JA, Kato K, *et al*. Nivolumab Combination Therapy in Advanced Esophageal Squamous-Cell Carcinoma. *N Engl J Med.* 2022;386(5):449-462.

114. Lee SM, Schulz C, Prabhash K, *et al*. First-line atezolizumab monotherapy versus single-agent chemotherapy in patients with non-small-cell lung cancer ineligible for treatment

with a platinum-containing regimen (IPSOS): a phase 3, global, multicentre, open-label, randomised controlled study. *Lancet.* 2023;402(10400):451-463.

115. Mittendorf EA, Zhang H, Barrios CH, *et al*. Neoadjuvant atezolizumab in combination with sequential nab-paclitaxel and anthracycline-based chemotherapy versus placebo and chemotherapy in patients with early-stage triple-negative breast cancer (IMpassion031): a randomised, double-blind, phase 3 trial. *Lancet*. 2020;396(10257):1090-1100.

116. Kaseb AO, Hasanov E, Cao HST, *et al*. Perioperative nivolumab monotherapy versus nivolumab plus ipilimumab in resectable hepatocellular carcinoma: a randomised, open-label, phase 2 trial. *Lancet Gastroenterol Hepatol.* 2022;7(3):208-218.

117. Tewari KS, Monk BJ, Vergote I, *et al*. Survival with Cemiplimab in Recurrent Cervical Cancer. *N Engl J Med*. 2022;386(6):544-555.

118. Boyer M, Şendur MAN, Rodríguez-Abreu D, *et al*. Pembrolizumab Plus Ipilimumab or Placebo for Metastatic Non-Small-Cell Lung Cancer With PD-L1 Tumor Proportion Score ≥ 50%: Randomized, Double-Blind Phase III KEYNOTE-598 Study. *J Clin Oncol.* 2021;39(21): 2327-2338.

119. Zhou C, Huang D, Fan Y, *et al*. Tislelizumab Versus Docetaxel in Patients With Previously Treated Advanced NSCLC (RATIONALE-303): A Phase 3, Open-Label, Randomized Controlled Trial. *J Thorac Oncol.* 2023;18(1):93-105.

120. Gogishvili M, Melkadze T, Makharadze T, *et al*. Cemiplimab plus chemotherapy versus chemotherapy alone in non-small cell lung cancer: a randomized, controlled, double-blind phase 3 trial. *Nat Med.* 2022;28(11):2374-2380.

121. Shen L, Kato K, Kim SB, *et al*. Tislelizumab Versus Chemotherapy as Second-Line Treatment for Advanced or Metastatic Esophageal Squamous Cell Carcinoma (RATIONALE-302): A Randomized Phase III Study. *J Clin Oncol.* 2022;40(26):3065-3076.

122. Finn RS, Qin S, Ikeda M, *et al*. Atezolizumab plus Bevacizumab in Unresectable Hepatocellular Carcinoma. *N Engl J Med*. 2020;382(20):1894-1905.

123. Rosenberg JE, Park SH, Kozlov V, *et al*. Durvalumab Plus Olaparib in Previously Untreated, Platinum-Ineligible Patients With Metastatic Urothelial Carcinoma: A Multicenter, Randomized, Phase II Trial (BAYOU). *J Clin Oncol*. 2023;41(1):43-53.

124. Tawbi HA, Schadendorf D, Lipson EJ, *et al*. Relatlimab and Nivolumab versus Nivolumab in

Untreated Advanced Melanoma. *N Engl J Med.* 2022;386(1):24-34.

125. Makker V, Colombo N, Casado Herráez A, *et al*. Lenvatinib plus Pembrolizumab for Advanced Endometrial Cancer. *N Engl J Med.* 2022;386(5):437-448.

126. Luke JJ, Rutkowski P, Queirolo P, *et al*. Pembrolizumab versus placebo as adjuvant therapy in completely resected stage IIB or IIC melanoma (KEYNOTE-716): a randomised, double-blind, phase 3 trial. *Lancet.* 2022;399(10336):1718-1729.

127. Cho BC, Abreu DR, Hussein M, *et al*. Tiragolumab plus atezolizumab versus placebo plus atezolizumab as a first-line treatment for PD-L1-selected non-small-cell lung cancer (CITYSCAPE): primary and follow-up analyses of a randomised, double-blind, phase 2 study. *Lancet Oncol.* 2022;23(6):781-792.

128. Mai HQ, Chen QY, Chen D, *et al*. Toripalimab or placebo plus chemotherapy as first-line treatment in advanced nasopharyngeal carcinoma: a multicenter randomized phase 3 trial. *Nat Med.* 2021;27(9):1536-1543.

129. Yang Y, Wang Z, Fang J, *et al*. Efficacy and Safety of Sintilimab Plus Pemetrexed and Platinum as First-Line Treatment for Locally Advanced or Metastatic Nonsquamous NSCLC: a Randomized, Double-Blind, Phase 3 Study (Oncology pRogram by InnovENT anti-PD-1-11). *J Thorac Oncol.* 2020;15(10):1636-1646.

130. Zhou C, Wu L, Fan Y, *et al*. Sintilimab Plus Platinum and Gemcitabine as First-Line Treatment for Advanced or Metastatic Squamous NSCLC: Results From a Randomized, Double-Blind, Phase 3 Trial (ORIENT-12). *J Thorac Oncol.* 2021;16(9):1501-1511.

131. Colombo N, Dubot C, Lorusso D, *et al*. Pembrolizumab for Persistent, Recurrent, or Metastatic Cervical Cancer. *N Engl J Med.* 2021;385(20):1856-1867.

132. Lu S, Wang J, Yu Y, *et al*. Tislelizumab Plus Chemotherapy as First-Line Treatment for Locally Advanced or Metastatic Nonsquamous NSCLC (RATIONALE 304): A Randomized Phase 3 Trial. *J Thorac Oncol*. 2021;16(9):1512-1522.

133. Ren S, Chen J, Xu X, *et al*. Camrelizumab Plus Carboplatin and Paclitaxel as First-Line Treatment for Advanced Squamous NSCLC (CameL-Sq): A Phase 3 Trial. *J Thorac Oncol*. 2022; 17(4):544-557.

134. Luo H, Lu J, Bai Y, *et al*. Effect of Camrelizumab vs Placebo Added to Chemotherapy on Survival and Progression-Free Survival in Patients With Advanced or Metastatic Esophageal

Squamous Cell Carcinoma: The ESCORT-1st Randomized Clinical Trial. *JAMA*. 2021;326(10): 916-925.

135. Yang Y, Qu S, Li J, *et al*. Camrelizumab versus placebo in combination with gemcitabine and cisplatin as first-line treatment for recurrent or metastatic nasopharyngeal carcinoma (CAPTAIN-1st): a multicentre, randomised, double-blind, phase 3 trial. *Lancet Oncol*. 2021;22(8): 1162-1174.

136. Wang J, Zhou C, Yao W, *et al*. Adebrelimab or placebo plus carboplatin and etoposide as first-line treatment for extensive-stage small-cell lung cancer (CAPSTONE-1): a multicentre, randomised, double-blind, placebo-controlled, phase 3 trial. *Lancet Oncol*. 2022;23(6):739-747.

137. Lu Z, Wang J, Shu Y, *et al*. Sintilimab versus placebo in combination with chemotherapy as first line treatment for locally advanced or metastatic oesophageal squamous cell carcinoma (ORIENT-15): multicentre, randomised, double blind, phase 3 trial. *BMJ*. 2022;377:e068714.

138. Xu J, Kato K, Raymond E, *et al*. Tislelizumab plus chemotherapy versus placebo plus chemotherapy as first-line treatment for advanced or metastatic oesophageal squamous cell carcinoma (RATIONALE-306): a global, randomised, placebo-controlled, phase 3 study. *Lancet Oncol*. 2023;24(5):483-495.

139. Zhou C, Wang Z, Sun Y, *et al*. Sugemalimab versus placebo, in combination with platinum-based chemotherapy, as first-line treatment of metastatic non-small-cell lung cancer (GEMSTONE-302): interim and final analyses of a double-blind, randomised, phase 3 clinical trial. *Lancet Oncol*. 2022;23(2):220-233.

140. Lu S, Wu L, Jian H, *et al*. Sintilimab plus bevacizumab biosimilar IBI305 and chemotherapy for patients with EGFR-mutated non-squamous non-small-cell lung cancer who progressed on EGFR tyrosine-kinase inhibitor therapy (ORIENT-31): first interim results from a randomised, double-blind, multicentre, phase 3 trial. *Lancet Oncol*. 2022;23(9):1167-1179.

141. Wang ZX, Cui C, Yao J, *et al*. Toripalimab plus chemotherapy in treatment-naïve, advanced esophageal squamous cell carcinoma (JUPITER-06): A multi-center phase 3 trial. *Cancer Cell*. 2022;40(3):277-288.e3.

142. Antonarakis ES, Park SH, Goh JC, *et al*. Pembrolizumab Plus Olaparib for Patients With Previously Treated and Biomarker-Unselected Metastatic Castration-Resistant Prostate Cancer: The Randomized, Open-Label, Phase III KEYLYNK-010 Trial. *J Clin Oncol*. 2023;41(22):

3839-3850.

143. Eskander RN, Sill MW, Beffa L, *et al*. Pembrolizumab plus Chemotherapy in Advanced Endometrial Cancer. *N Engl J Med.* 2023;388(23):2159-2170.

144. Hu H, Kang L, Zhang J, *et al*. Neoadjuvant PD-1 blockade with toripalimab, with or without celecoxib, in mismatch repair-deficient or microsatellite instability-high, locally advanced, colorectal cancer (PICC): a single-centre, parallel-group, non-comparative, randomised, phase 2 trial. *Lancet Gastroenterol Hepatol.* 2022;7(1):38-48.

145. Fu Q, Chen Y, Huang D, *et al*. Sintilimab Plus Modified FOLFIRINOX in Metastatic or Recurrent Pancreatic Cancer: The Randomized Phase II CISPD3 Trial. *Ann Surg Oncol.* 2023; 30(8):5071-5080.

146. Kelley RK, Ueno M, Yoo C, *et al*. Pembrolizumab in combination with gemcitabine and cisplatin compared with gemcitabine and cisplatin alone for patients with advanced biliary tract cancer (KEYNOTE-966): a randomised, double-blind, placebo-controlled, phase 3 trial. *Lancet.* 2023;401(10391):1853-1865.

147. Pal SK, Albiges L, Tomczak P, *et al*. Atezolizumab plus cabozantinib versus cabozantinib monotherapy for patients with renal cell carcinoma after progression with previous immune checkpoint inhibitor treatment (CONTACT-03): a multicentre, randomised, open-label, phase 3 trial. *Lancet.* 2023;402(10397):185-195.

Supplemental file 4

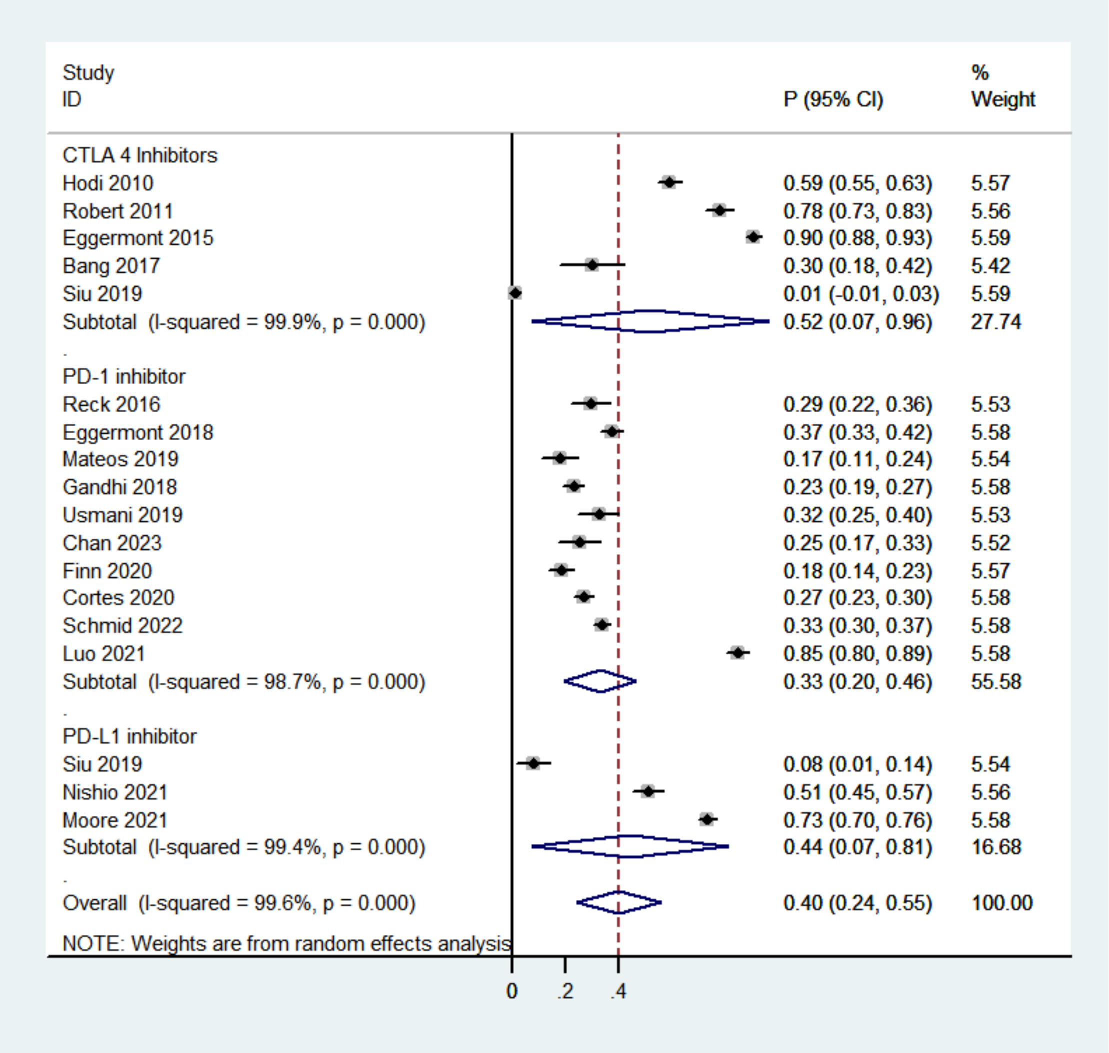

Figure S1. The incidences of all-grade irAEs according to the type of ICI

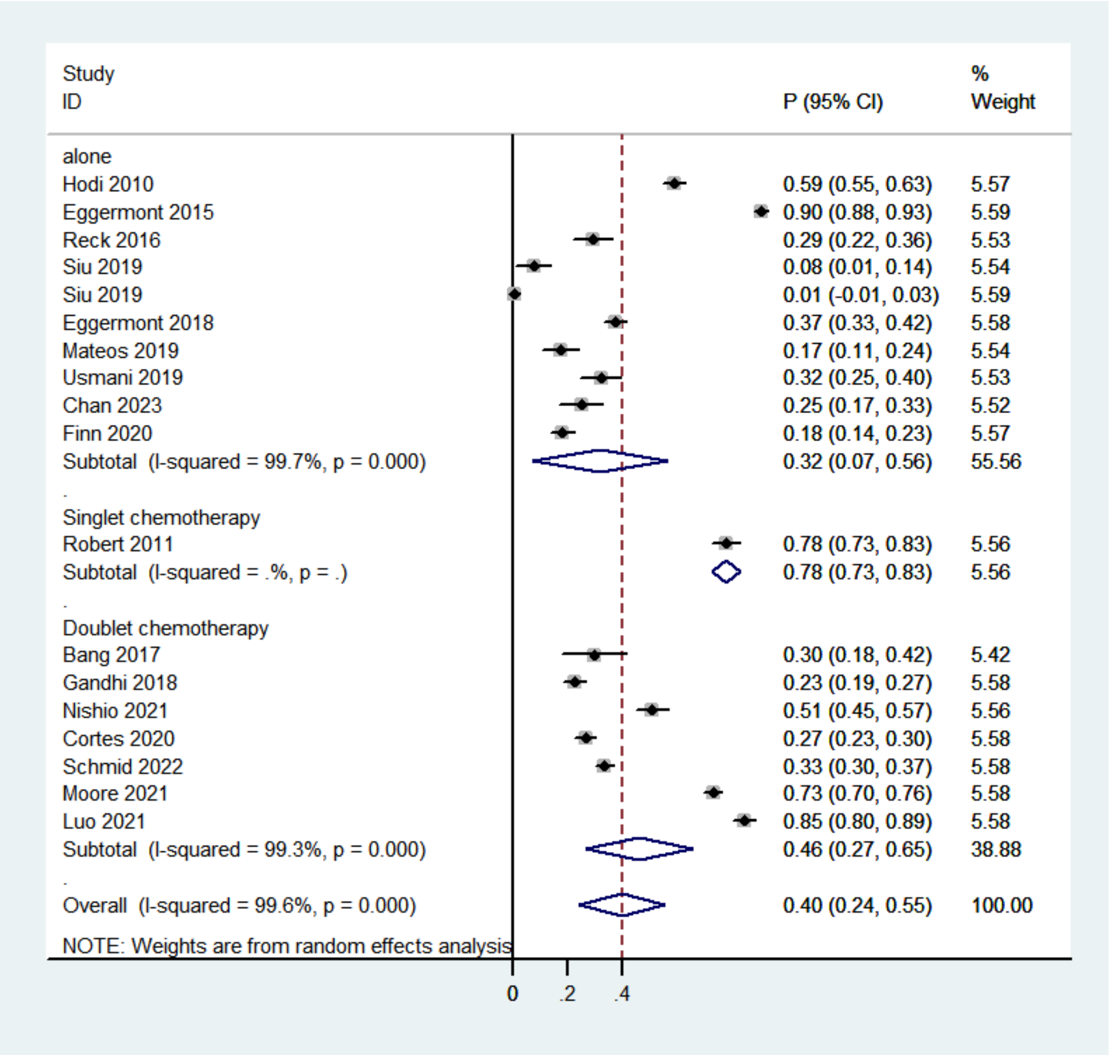

Figure S2. The incidences of all-grade irAEs according to the combination therapy

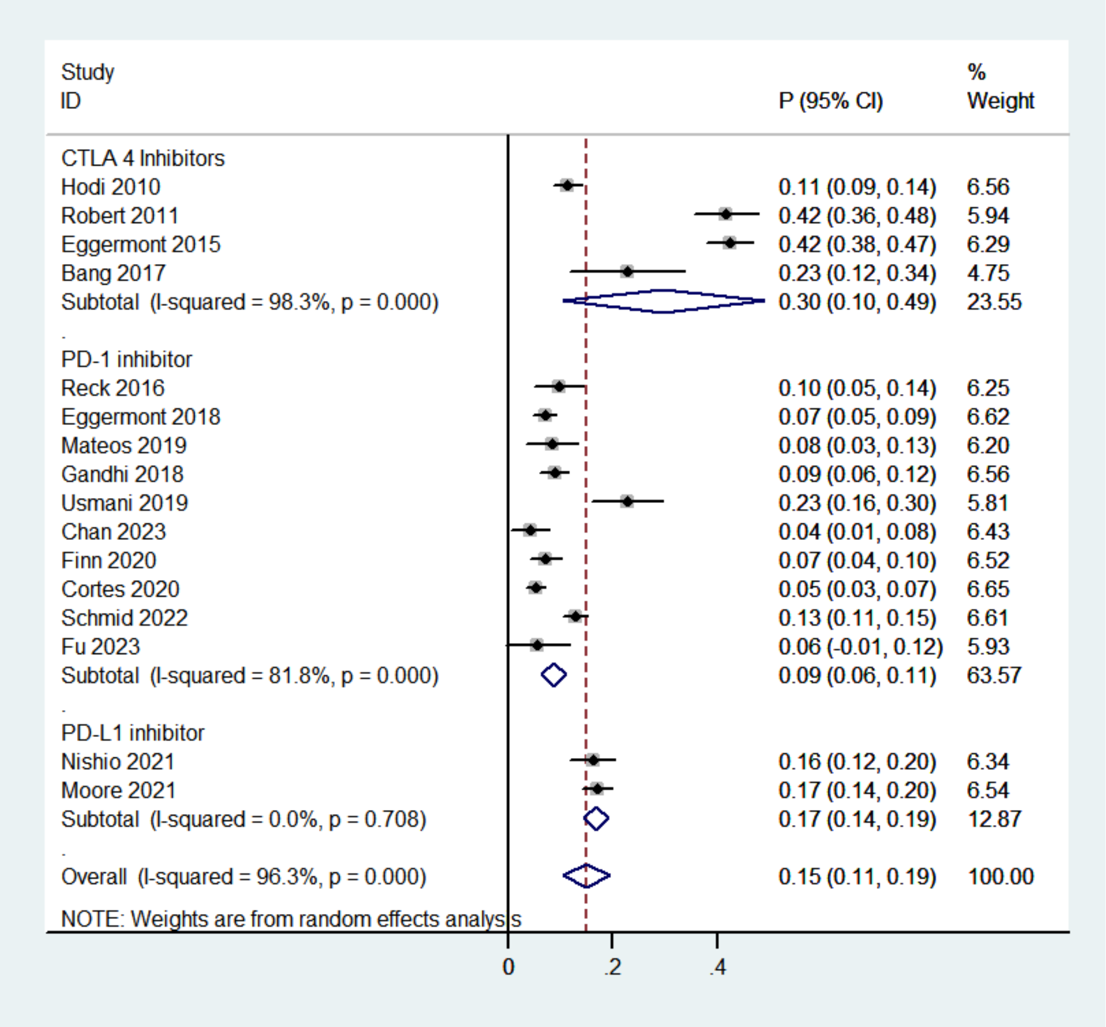

Figure S3. The incidences of grade ≥ 3 irAEs according to the type of ICI

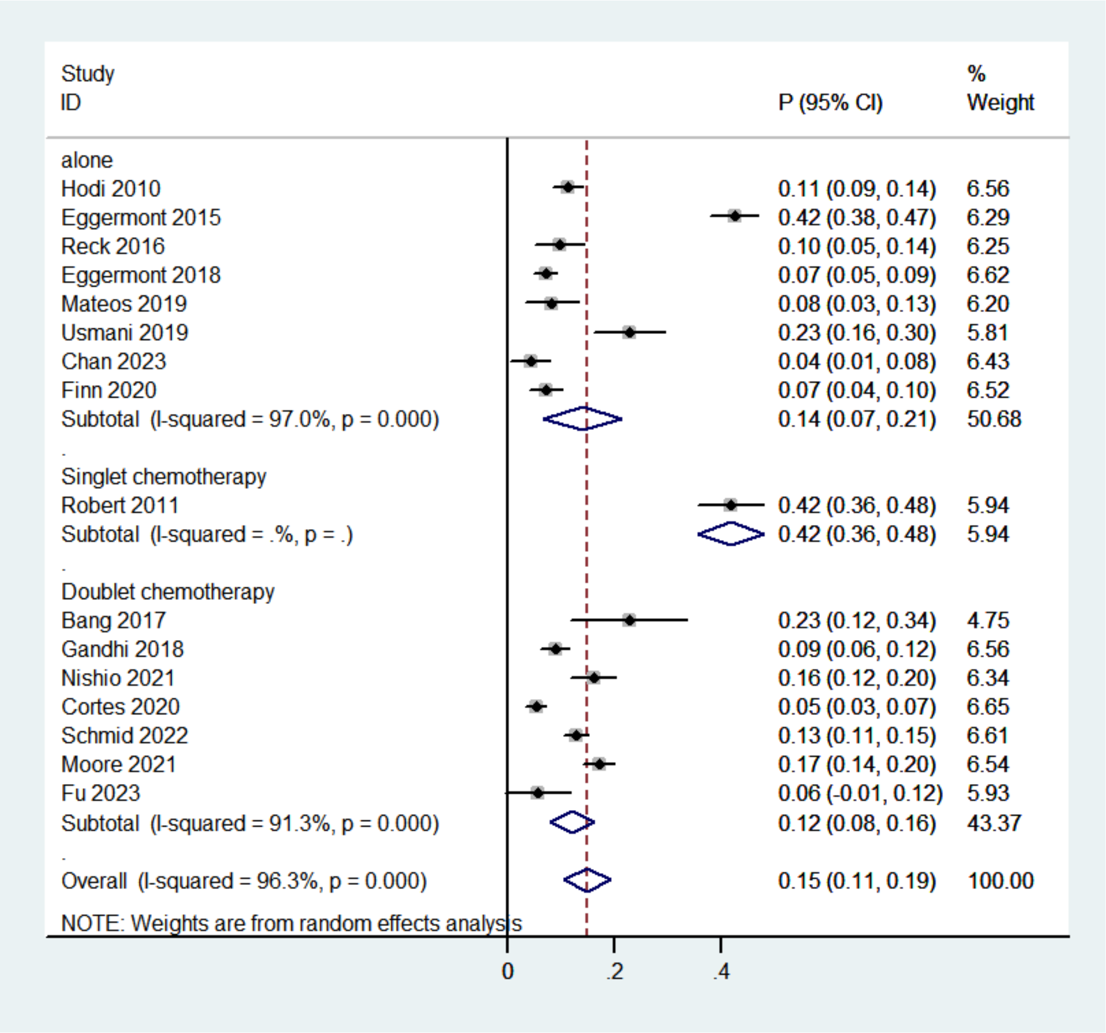

Figure S4. The incidences of grade ≥ 3 irAEs according to the combination therapy

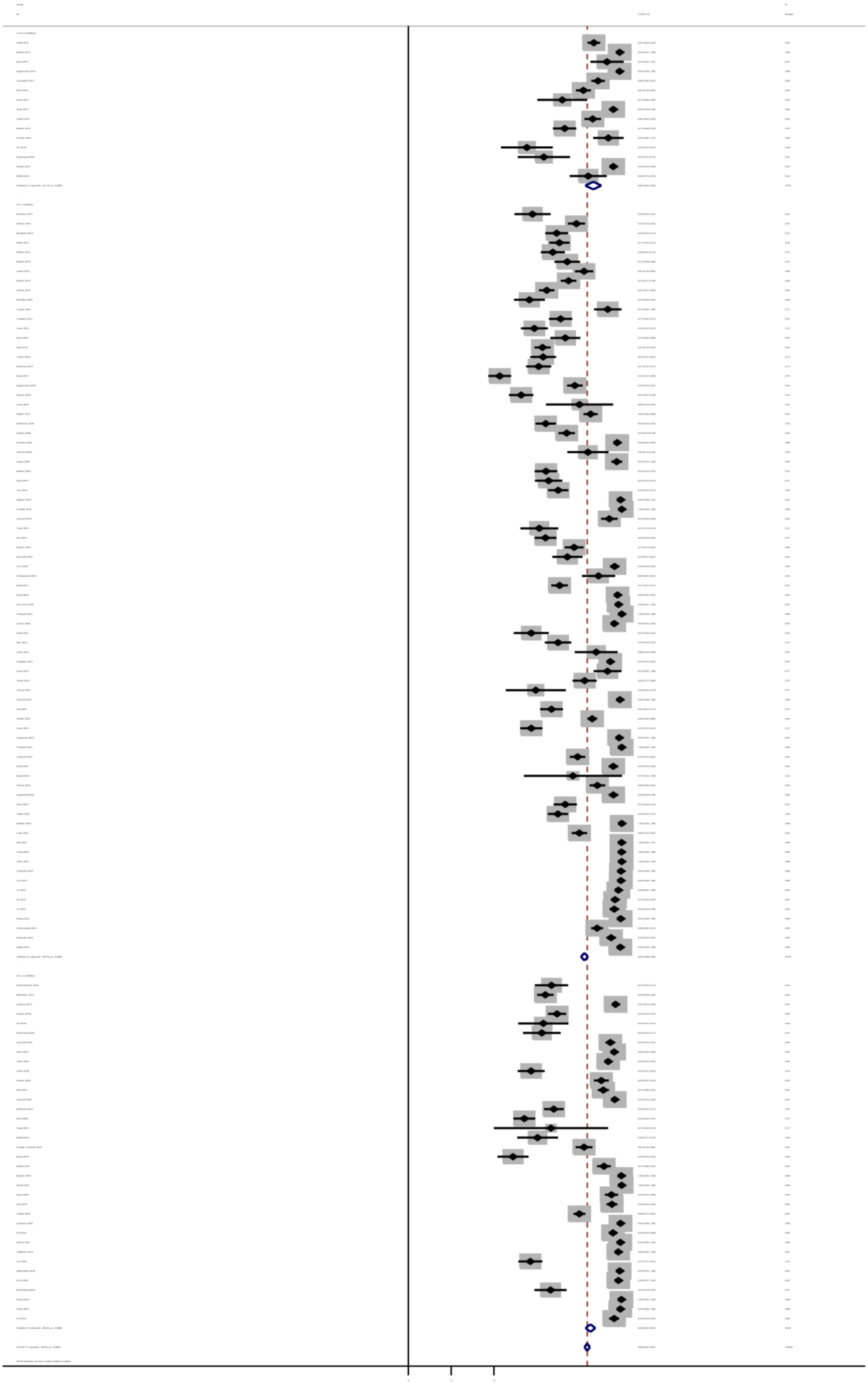

Figure S5. The incidences of all-grade trAEs according to the type of ICI

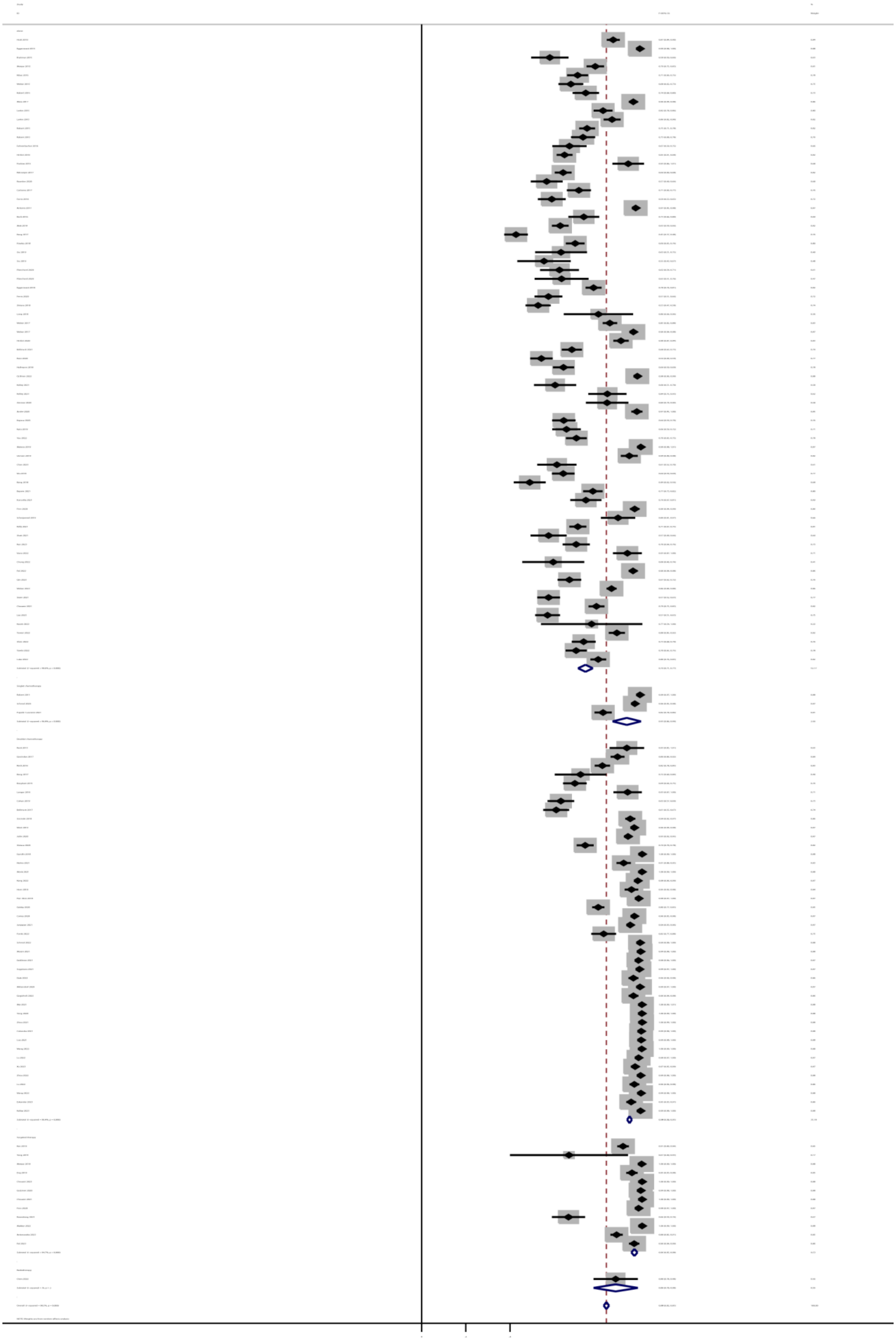

Figure S6. The incidences of all-grade trAEs according to the combination therapy

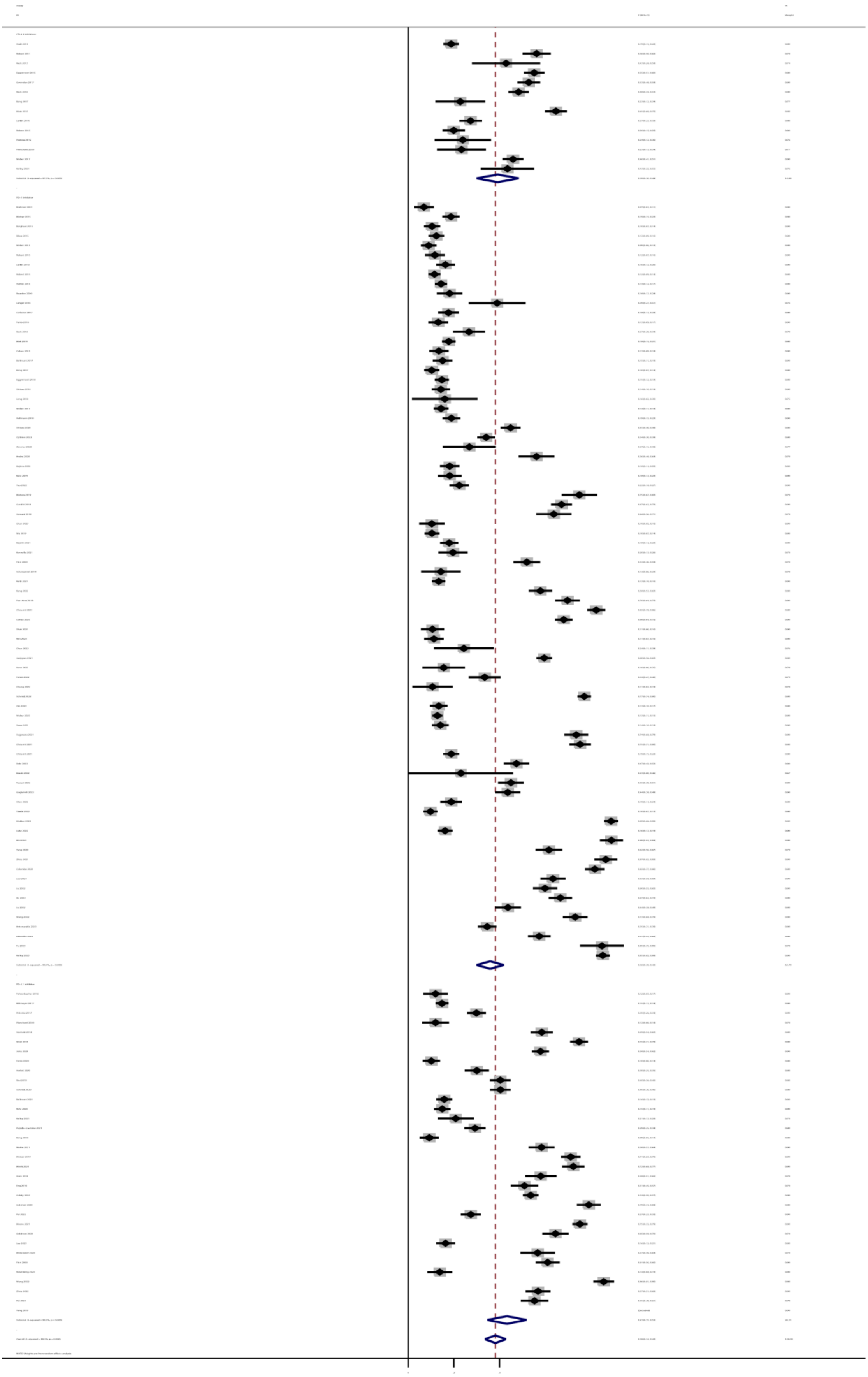

Figure S7. The incidences of grade ≥ 3 trAEs according to the type of ICI

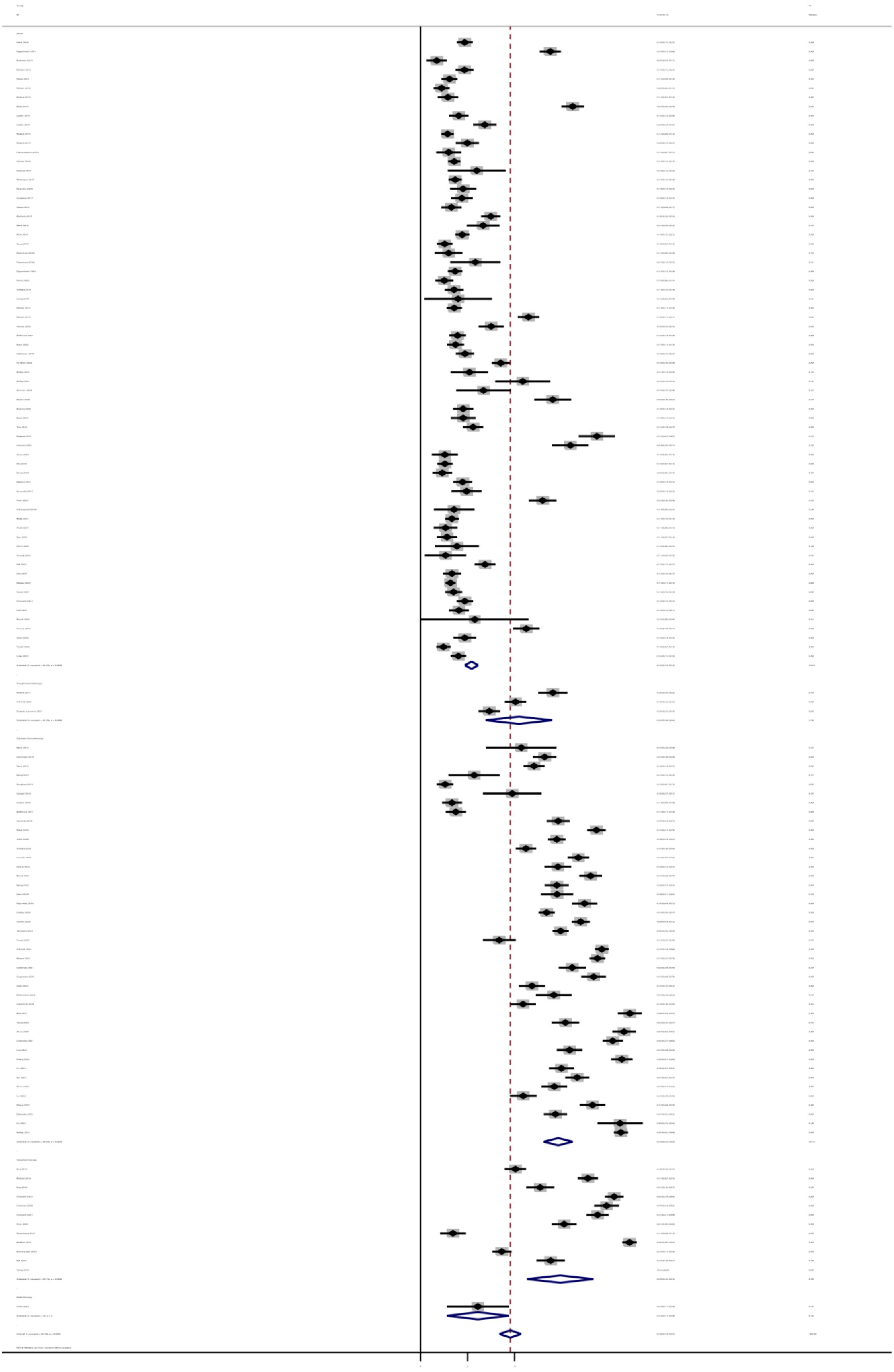

Figure S8. The incidences of grade ≥ 3 trAEs according to the combination therapy

Supplemental file 5

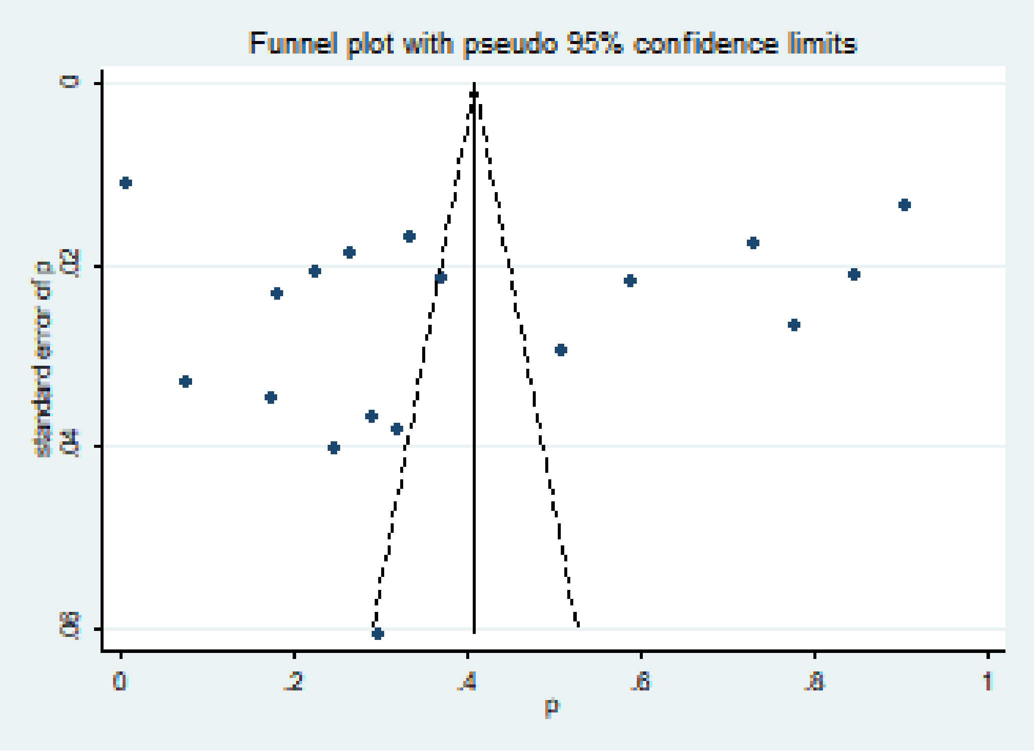

Figure S1. Funnel plot for the incidences of all-grade irAEs (P value for Egger: 0.870; P value for Begg: 0.762)

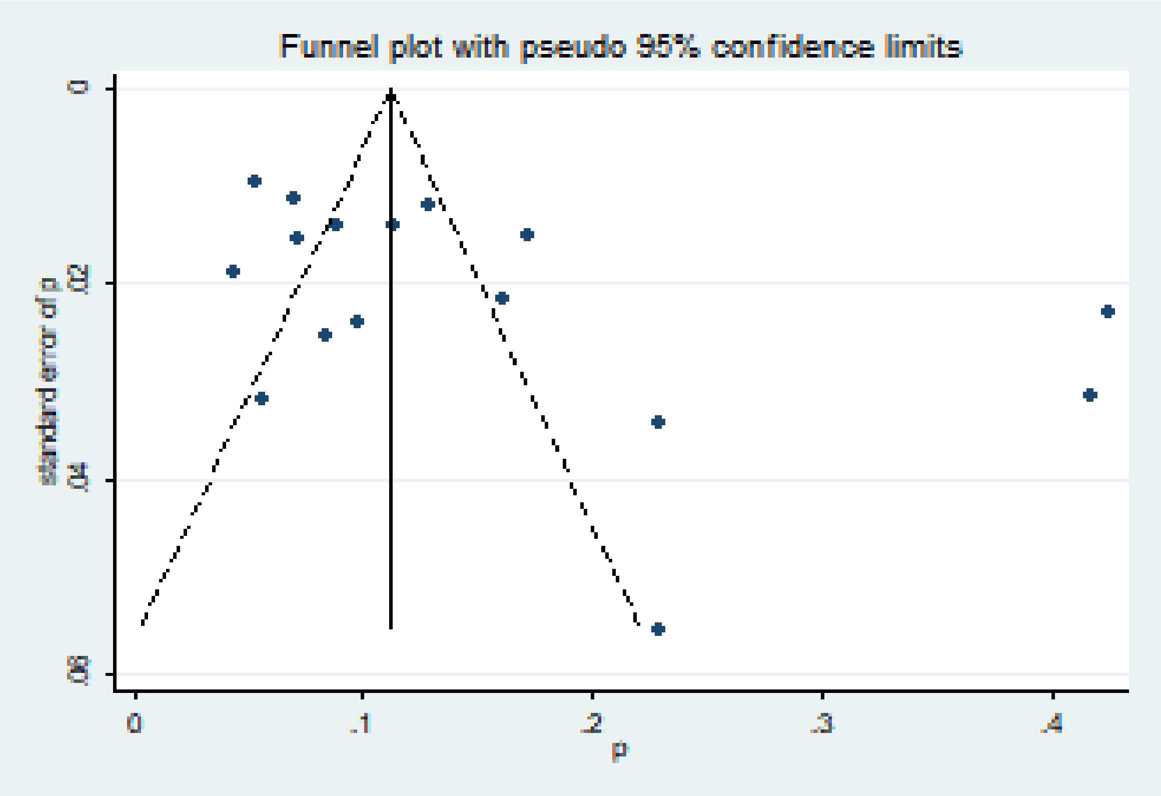

Figure S2. Funnel plot for the incidences of grade ≥ 3 irAEs (P value for Egger: 0.045; P value for Begg: 0.115)

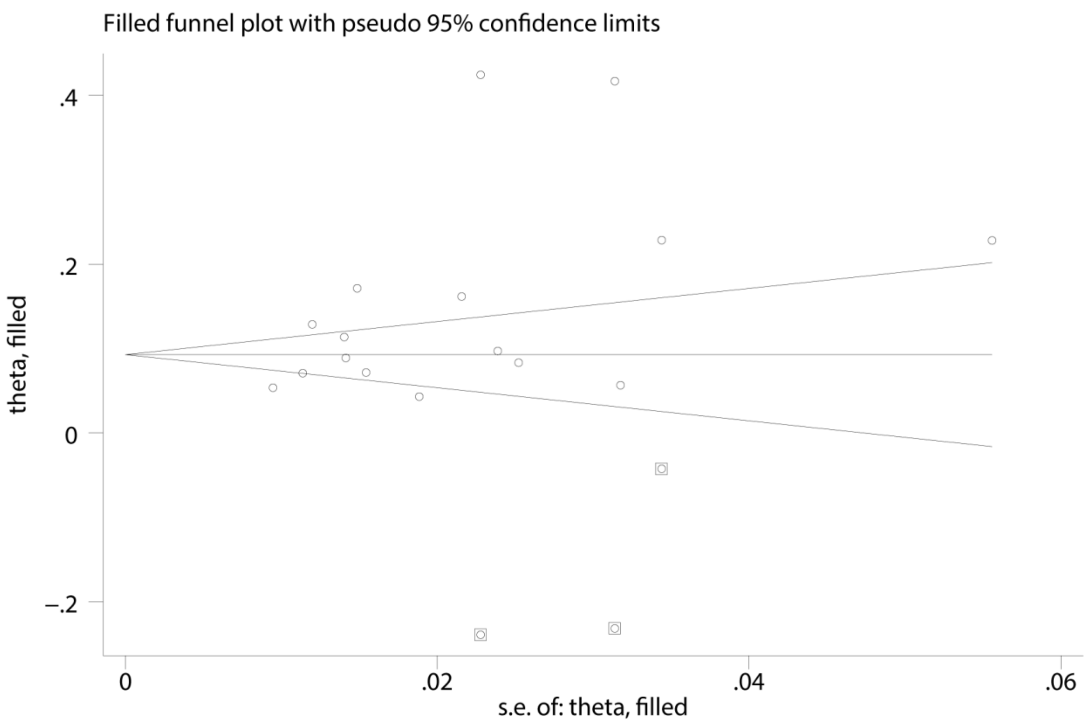

Figure S3. Trim and fill method for the incidences of grade ≥ 3 irAEs (10.0%; 95%CI: 4.8%-15.3%)

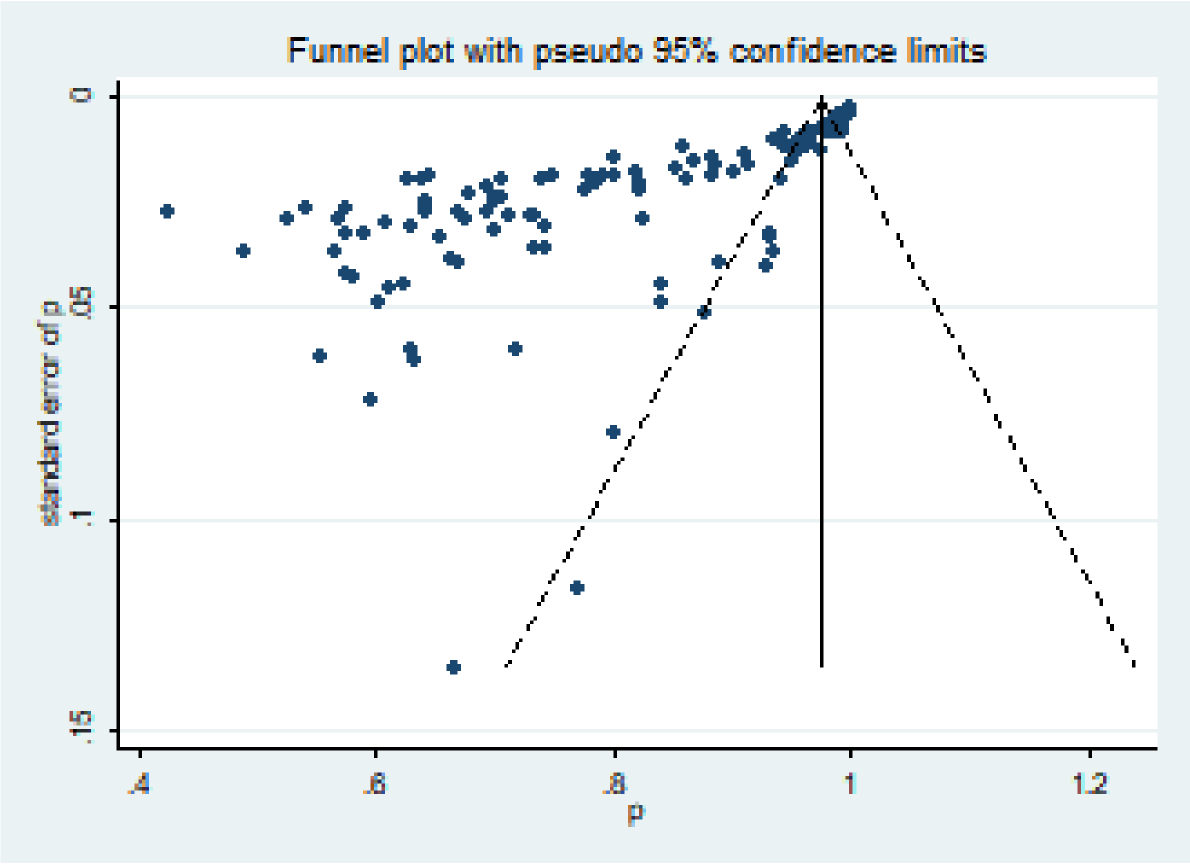

Figure S4. Funnel plot for the incidences of all-grade trAEs (P value for Egger: < 0.001; P value for Begg: < 0.001)

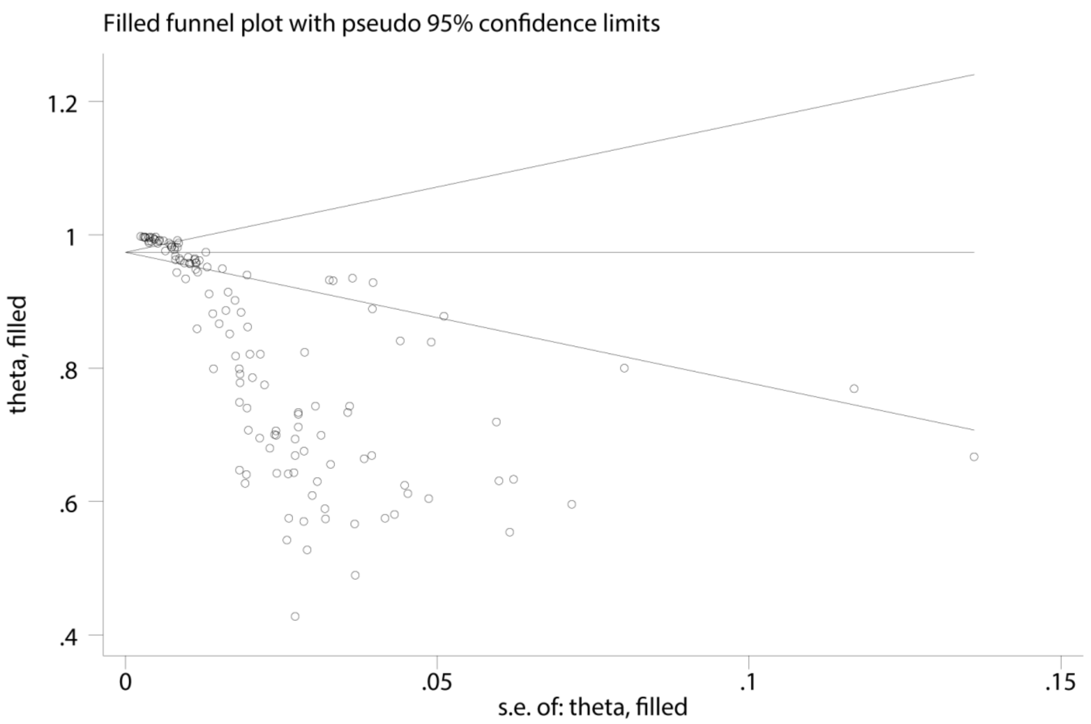

Figure S5. Trim and fill method for the incidences of all-grade trAEs (83.6%; 95%CI: 82.4%-84.8%)

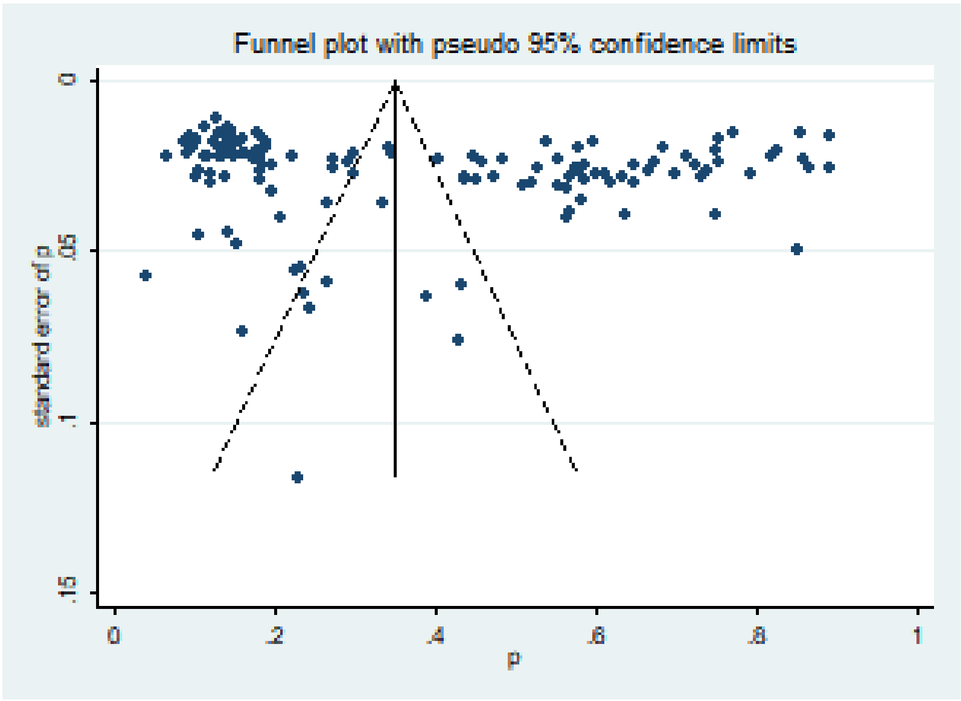

Figure S6. Funnel plot for the incidences of grade ≥ 3 trAEs (P value for Egger: 0.023; P value for Begg: < 0.001)

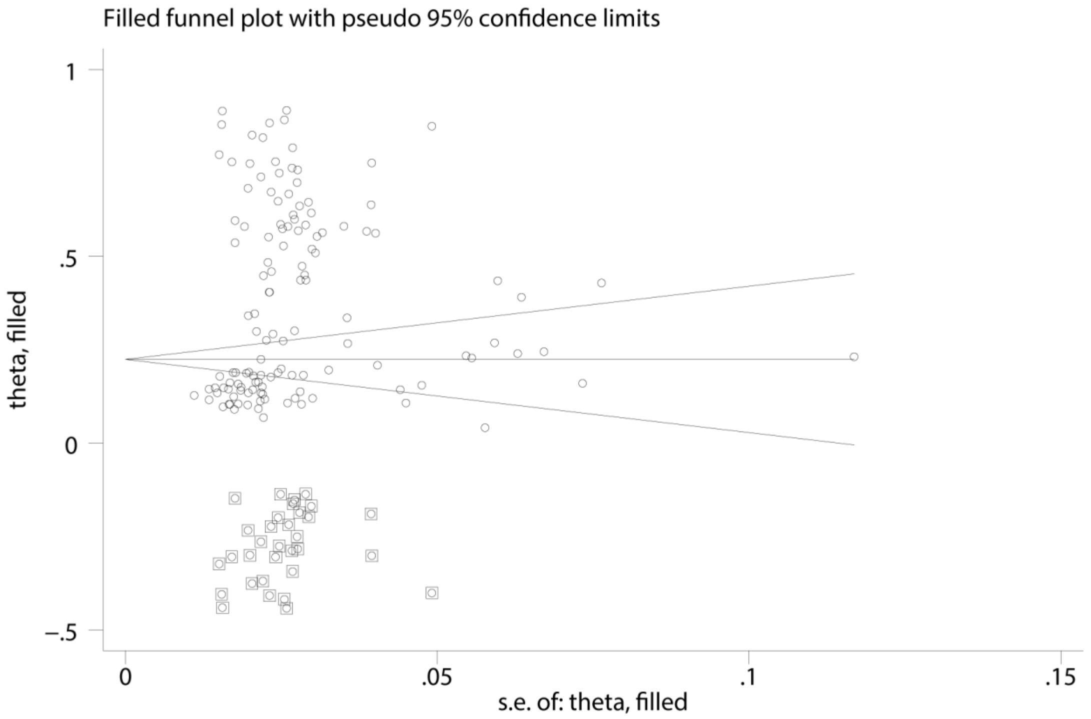

Figure S7. Trim and fill method for the incidences of grade ≥ 3 trAEs (24.7%; 95%CI: 19.2%-30.1%)
